# Exposure to air pollutants and breast cancer risk: mediating effects of metabolic health biomarkers in a nested case–control study within the E3N-Generations cohort

**DOI:** 10.1186/s13058-024-01913-7

**Published:** 2024-11-15

**Authors:** Benoît Mercoeur, Béatrice Fervers, Thomas Coudon, Hwayoung Noh, Camille Giampiccolo, Lény Grassot, Elodie Faure, Florian Couvidat, Gianluca Severi, Francesca Romana Mancini, Pascal Roy, Delphine Praud, Amina Amadou

**Affiliations:** 1 Department of Prevention Cancer Environnement, Centre Léon Bérard, Lyon, France; 2 Inserm U1296 Radiations : Défense, Santé, Environnement, Lyon, France; 3 Laboratoire de Biométrie Et Biologie Evolutive, CNRS UMR 5558, Villeurbanne, France; 4 Service de Biostatistique-Bioinformatique, Pole Sante Publique, Hospices Civils de Lyon, Lyon, France; 5 Universite Paris-Saclay, UVSQ, Inserm, Gustave Roussy, CESP, 94805 Villejuif, France; 6 National Institute for Industrial Environment and Risks (INERIS), Verneuil-en-Halatte, France; 7 Department of Statistics, Computer Science and Applications (DISIA), University of Florence, Florence, Italy; 8 Université Claude Bernard Lyon 1, Lyon, France

**Keywords:** Breast cancer, Air pollutants, Biomarkers, Mediation, Interaction

## Abstract

**Background:**

Growing epidemiological evidence suggests an association between exposure to air pollutants and breast cancer. Yet, the underlying mechanisms remain poorly understood. This study explored the mediating role of thirteen metabolic health biomarkers in the relationship between exposure to three air pollutants, i.e. nitrogen dioxide (NO_2_), polychlorinated biphenyls 153 (PCB153), and benzo[a]pyrene (BaP), and breast cancer risk.

**Methods:**

We used data from a nested case–control study within the French national prospective E3N-Generations cohort, involving 523 breast cancer cases and 523 matched controls. The four-way decomposition mediation of total effects for thirteen biomarkers was applied to estimate interaction and mediation effects (controlled direct, reference interaction, mediated interaction, and pure indirect effects).

**Results:**

The analyses indicated a significant increase in breast cancer risk associated with BaP exposure (odds ratio (OR)_Q4 vs Q1_ = 2.32, 95% confidence intervals (CI): 1.00–5.37). PCB153 exposure showed a positive association only in the third quartile (OR_Q3 vs Q1_ = 2.25, CI 1.13–4.57), but it appeared to be non-significant in the highest quartile (OR_Q4 vs Q1_ = 2.07, CI 0.93–4.61). No association was observed between NO_2_ exposure and breast cancer risk. Estradiol was associated with an increased risk of breast cancer (OR per one standard deviation (SD) increment = 1.22, CI 1.05–1.42), while thyroid-stimulating hormone was inversely related to breast cancer risk (OR per 1SD increase = 0.87, CI 0.75–1.00). We observed a suggestive mediated effect of the association between the three pollutants and breast cancer risk, through albumin, high-density lipoproteins cholesterol, low-density lipoprotein cholesterol, parathormone, and estradiol.

**Conclusion:**

Although limited by a lack of statistical power, this study provides relevant insights into the potential mediating role of certain biomarkers in the association between air pollutant exposure and breast cancer risk, highlighting the need for further in-depth studies in large populations.

**Supplementary Information:**

The online version contains supplementary material available at 10.1186/s13058-024-01913-7.

## Background

Outdoor air pollution, a complex mixture of atmospheric pollutants including gases, particles, metals, and organic compounds, is a major contributor to global mortality [1].The International Agency for Research on Cancer (IARC) classified outdoor air pollution as a whole as carcinogenic in humans [2]. Among these, nitrogen dioxide (NO_2_) is a common pollutant that has been linked to various adverse health effects, including breast cancer development [3, 4]. NO_2_ is primarily emitted from the combustion of fossil fuels (heating, power generation) and motor vehicles [5]. Polychlorinated biphenyls (PCB) and benzo[a]pyrene (BaP) are two endocrine-disrupting pollutants (EDP) that are associated with an increased incidence of numerous diseases, notably breast cancer risk [6–8]. These compounds are mainly emitted from industrial activities and biomass combustion [7, 9].

Recent epidemiological studies increasingly link air pollution to breast cancer, suggesting statistically significant relationships between certain air pollutants and an increased risk of breast cancer, though the findings are not entirely inconsistent [10–13]. NO_2_ has been associated with a higher risk of breast cancer in case-control studies [14, 15]. A recent meta-analysis further supported this association, indicating NO_2_ as a common marker of traffic-related air pollutants (TRAP) linked to breast cancer [16]. Similarly, elevated levels of BaP and polycyclic aromatic hydrocarbons (PAHs) have been associated with increased breast cancer risk [17, 18], and PCBs (including PCB153) have shown a positive association with breast cancer across several epidemiological studies, including meta-analyses [7, 19, 20].

Although not fully elucidated, several biological mechanisms that might explain how these pollutants are involved in the development of breast cancer have been proposed. EDP can bind to estrogen receptors [21] and the aryl hydrocarbon receptor [22, 23], activating pathways involved in the carcinogenesis. These EDP increase levels of endogenous hormone levels [24], particularly estrogen and progesterone, which are directly linked to breast cancer [25]. Sex Hormone-Binding Globulin (SHBG) also plays a crucial role in the pathophysiology of breast cancer, primarily by regulating circulating estradiol [26]. Consequently, a decrease in SHBG levels is associated with a higher risk of breast cancer development. Additionally, androgens, such as testosterone, significantly influence on breast cancer [27]. Moreover, exposure to the three air pollutants (NO_2_, BaP, and PCB153) can lead to a number of changes and perturbation as hallmarks in cancer development [28], including chronic inflammations through increase in blood levels of pro-inflammatory factors [29] and C-reactive protein (CRP) [30], and disturbances in lipid metabolism, such as elevated cholesterol levels [31, 32].

Yet, the precise roles of these biomarkers, considering their interacting and mediating effects in the associations between the air pollutants of interest and breast cancer, remain unclear. A mediation approach, which considers both mediation and interaction, is a valuable tool for better understanding the underlying mechanisms and unravelling the different pathways of the association of air pollutants with breast cancer. Mediation analysis is generally applied to evaluate to what extent the effect of an exposure is explained or not, by a set of hypothetical mediators. In recent years, integrating causal inference approaches has significantly advanced mediation analysis, resulting in more robust and generalizable methods for understanding direct and indirect effects [33].

The objective of the present study was, therefore, to explore the mediating role of various biomarkers of metabolic health in the relationship between three air pollutants (NO_2_, BaP and PCB153) and risk of breast cancer using a four-way decomposition mediation analysis [34].

## Methods

### Study population

The present study was conducted using a sub-sample of 523 breast cancer cases and 523 matched controls from the XENAIR study [35], for whom measurements of biomarkers were available. This nested case-control study within the national E3N-Generations cohort, included 5,222 cases of invasive breast cancer and 5,222 matched controls followed from 1990 (at baseline) to 2011 [17, 20]. As described in our previous studies, controls were randomly selected from women who were free of breast cancer, based on incidence density sampling and matched to controls according to age, date, menopausal status, residential area, and blood sample [35]. The flowchart of study participants selection is provided in the Supplementary Fig. 1.

The E3N-Generations prospective study, a continuing French cohort study, was established as an extension of the E3N cohort of women (Etude Epidémiologique auprès des femmes de la Mutuelle Générale de l’Education Nationale), which includes the E3N women’s children, their fathers and, in the future, their grandchildren.

The E3N-cohort Generations 1 was started in 1990 to investigate the key risk factors for cancer and chronic diseases among women [36]. At recruitment (1990-1991), a total of 98,995 French women aged 40 to 65 years old, and insured with MGEN (a national health insurance scheme covering primarily teachers) were recruited. Participants completed self-administered questionnaires that collect data on socio-demographic characteristics, lifestyle, reproductive factors, anthropometry, past medical history, and familial history of cancer. The addresses of the cohort participants were collected at baseline and at each of the thirteen follow-ups questionnaires. Self-reported cases were validated through the retrieval of medical records from treating physicians, with pathological confirmation received for 93% of cases. The study was approved by the French National Commission for Data Protection and Privacy (CNIL), and informed consent was obtained from each participant.

### Pollutant exposure assessment

As previously described [14], long-term exposure levels of the three pollutants (NO_2_, BaP and PCB153) were estimated at the subjects’ residential addresses using two models in accordance of the existence of measurement and emission data of the pollutants of interest for the study period (1990-2011).

BaP and PCB153 were estimated using is a chemistry-transport model “CHIMERE”. This model, with a spatial resolution of 0.125° × 0.0625° (around 7 × 7 km) simulates pollutant transport from local to continental scales, by utilizing data (e.g. emission, meteorological fields, and boundary conditions) as inputs and runs a set of equations reflecting the physicochemical steps associated with the evolution of concentrations [37]. CHIMERE takes into account main particles that are directly emitted and whether they are anthropogenic or natural, and models the concentrations levels of each particle with aerodynamic diameters varying from a few nanometers to 10 μm [37]. NO_2_ levels were evaluated using a land use regression (LUR, 50 × 50 m) model, a widely used approach to model and to predict spatial variations in air pollution concentrations [38, 39]. The model employs proximity measures like circular buffers of different sizes, to capture geographical features that explain variability in monitored concentrations at specific locations (i.e. monitoring sites or addresses) [40, 41]. In the present study, a LUR model (50 × 50 m) was developed using the average annual NO_2_ data for the period of 2010 to 2012 [14]. This “baseline” model further incorporated inputs from COPERNIC (a chemical transport model providing NO_2_ background concentrations across France) and localised variables related to road traffic and land use, available throughout the country[41, 42]. The model underwent validation through comparisons with measurements across France using a hold-out validation approach with independent monitoring sites. The LUR model was retrospectively extrapolated to 1990 using annual local trends derived from the CHIMERE model [43].

For each woman, annual mean concentration of NO_2_, BaP and PCB153 were evaluated at their geocoded residential addresses for each year from 1990 to 2011. The average of these annual mean concentration for each pollutant were then calculated for each woman from the year they entered into the cohort until their index date (which corresponds to the date of breast cancer diagnosis for cases and date of selection for controls).

### Metabolic health biomarker assays

Biomarker levels were measured from the cohort blood samples collected between 1995 and 1998 [36]. The biomarkers investigated in this study were chosen based on their previously established individual associations with breast cancer risk and air pollutants [21, 22, 24, 27, 29, 30, 44]. These included pre-diagnostic circulating levels of albumin (g/L), c-reactive protein (CRP) (mg/L), triglycerides (mmol/L), cholesterol (mmol/L), high-density lipoproteins cholesterol (HDL) (mmol/L), low-density lipoproteins cholesterol (LDL) (mmol/L), parathormone (PTH) (pg/mL), thyroid-stimulating hormone (mlU/L), prolactin (mIU/L), estradiol (pmol/L), testosterone (nmol/L), SHBG (nmol/L) and progesterone (nmol/L).

Albumin and CRP were quantified by bromocresol green (BCG) analysis and immunoturbidimetric-high sensitivity analysis, respectively, using a Hitachi 911 analyzer (Roche Diagnostics, US) [45]. Using a modular analyzer (Roche Diagnostics, US), triglycerides, cholesterol, HDL, and LDL were quantified employing enzyme immune-inhibition analysis [45]. PTH, thyroid-stimulating hormone, prolactin, estradiol, testosterone, SHBG and progesterone were quantified by electrochemiluminescence immunoassay (ECLIA) method using the Elecsys analyzer (Roche Diagnostics, US) [45].

### Statistical analysis

The main characteristics of the population and biomarker levels were described distinctly for cases and controls, using means, standard deviations (SDs), percentiles, minimum and maximum values for continuous variables, and counts and percentages for qualitative variables. Pearson correlation analyses were performed to check correlations between biomarkers. The linearity of the pollutant-cancer and mediator-cancer associations was verified using restricted cubic splines with four degrees of freedom [46]. Conditional logistic regressions were employed to calculate odds ratios (ORs) and their corresponding 95% confidence intervals (CIs) for the associations between exposure to each pollutant and the risk of breast cancer. We modelled the pollutants as continuous variables (one SD increase) and as categorical variables (quartiles). Linear regression analyses were used to estimate the associations between each pollutant level and each biomarker of metabolic health with adjustments for confounders. The effect of each biomarker on breast cancer (per one SD increase) was estimated using conditional logistic regression analyses.

**Fig. 1:**
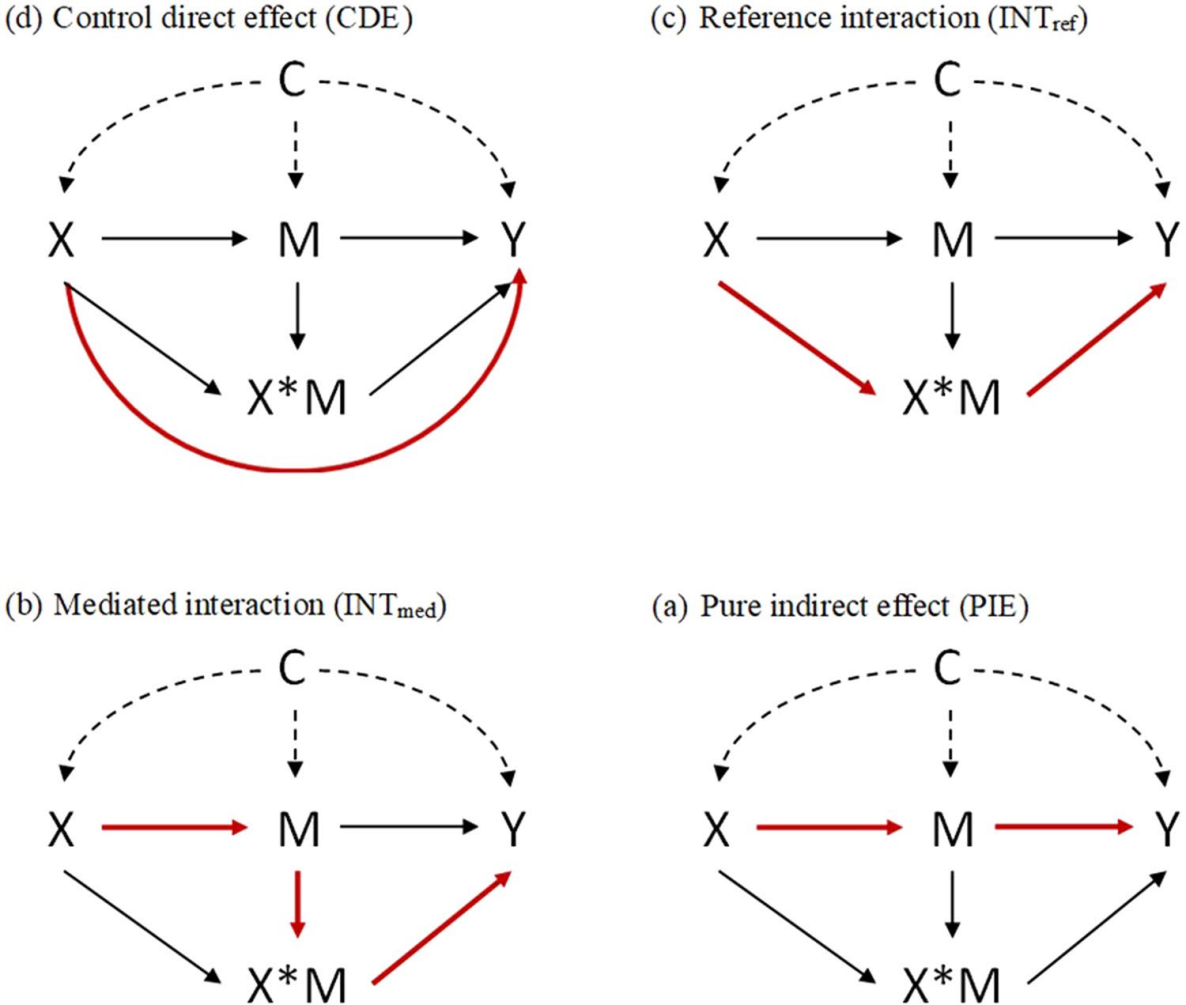
Causal diagram with the interaction representing a 4-way decomposition X: the exposure, M: the mediator, X × M: the interaction between the exposure and the mediator, Y: the outcome, C: a set of confounders. Red line shows each effect

A four-way decomposition mediation analysis was fitted to assess whether the associations between atmospheric pollutants and breast cancer risk were mediated by selected biomarkers [34]. Data on n individuals were observed as independent and identically distributed (C, X, M, Y), with Y being the binary outcome of interest, X the exposure, M a continuous mediator variable measured after X but before Y, and C representing pre-exposure confounders of the effects of (X, M) on Y. (Figure 1). The four-way decomposition analysis assumes that after adjusting for the potential confounders, there is no unobserved confounding that affects the relationship between exposure and outcome, and between exposure and mediator, and there are no confounders of the mediator-outcome relationship that may be affected by the exposure (post-exposure confounders) [47]. This approach allows us to determine the controlled direct effect (CDE), the reference interaction effect (INT_ref_), the mediated interaction effect (INT_med_) and the Pure Indirect Effect (PIE) (Fig. 1), assuming the following regression models:

1 $$\log it\left\{ {\Pr \left( {Y = 1|X = x,M = m,C = c} \right)} \right\} = \theta_{0} + \theta_{1} x + \theta_{2} m + \theta_{3} xm + \theta_{4}^{\prime} c$$

And

2 $$\begin{array}{c}E\left[M|X=x,C=c\right]={\beta }_{0}+{\beta }_{1x}+{\beta }_{2}^{\prime}c\end{array}$$

VanderWeele and Vansteelandt derived expressions for the CDE and the PIE all on the risk ratio scale. The total effect (TE), CDE, and PIE were given by:

3 $$\begin{array}{c}R{R}_{c}^{TE}=exp\left[{\theta }_{1}+{\theta }_{2}{\beta }_{1}+{\theta }_{3}\left({\beta }_{0}+{\beta }_{1}{x}^{*}+{\beta }_{1}x+{\beta }_{2}^{\prime}c+{\theta }_{2}{\sigma }^{2}\right)\left(x-{x}^{*}\right)+\frac{1}{2}{\theta }_{3}^{2}{\sigma }^{2}\left({x}^{2}-{x}^{*2}\right)\right]\end{array}$$

The control direct effect is given by:

4 $$\begin{array}{c}R{R}_{c}^{CDE}\left({m}^{*}\right)=exp\left[\left({\uptheta }_{1}+{\uptheta }_{3}{m}^{*}\right)\left(x-{x}^{*}\right)\right]\end{array}$$

The reference interaction is given by:

5 $$\begin{array}{c}R{R}_{c}^{IN{T}_{ref}}\left({m}^{*}\right)=\int \left\{\frac{E\left[x,m,c\right]}{E\left[{x}^{*},{m}^{*},c\right]}-\frac{E\left[{x}^{*},m,c\right]}{E\left[{x}^{*},{m}^{*},c\right]}-\frac{E\left[x,{m}^{*},c\right]}{E\left[{x}^{*},{m}^{*},c\right]}+1\right\}dP\left(m|{x}^{*}c\right)\end{array}$$

The mediated interaction is given by:

6 $$RR_{c}^{{INT_{med} }} = \smallint \left\{ {\frac{{E\left[ {x,m,c} \right]}}{{E\left[ {x^{*} ,m^{*} ,c} \right]}} - \frac{{E\left[ {x^{*} ,m,c} \right]}}{{E\left[ {x^{*} ,m^{*} ,c} \right]}}} \right\}\left\{ {dP\left( {x,c} \right) - dP\left( {x^{*} ,c} \right)} \right\}$$

The pure indirect effect is given by:

7 $$\begin{array}{c}R{R}_{c}^{PIE}=exp\left[\left({\uptheta }_{2}{\upbeta }_{1}+{\uptheta }_{3}{\upbeta }_{1}{x}^{*}\right)\left(x-{x}^{*}\right)\right]\end{array}$$

In this study, the CDE corresponds to the effect of the pollutant on breast cancer risk without mediation by the biomarker and without interaction between the pollutant and the biomarker. The INT_med_ corresponds to the effect of the pollutant on the breast cancer risk due to both the mediation of the biomarker and the interaction between the pollutant and the biomarker. The INT_ref_ corresponds to the effect of the pollutant on the breast cancer risk due solely to the interaction between the pollutant and the biomarker. The PIE corresponds to the effect of the pollutant on the breast cancer risk due solely to the mediation by the biomarker.

The sum of these four effects (i.e. CDE, INT_ref_, INT_med_, PIE) equals the total effect (TE) of the pollutant on breast cancer risk. The proportion of each of the four effects is calculated relative to the TE, thus, their sum equals 1. Of note, in some situations, negative proportions and proportions exceeding 100% may be observed. A negative proportion indicates that the indirect effect is opposite to the TE. In this case, the proportions of other effects may exceed 100%. This scenario typically arises when the associations between exposure and biomarker, and between biomarker and outcome are in opposite directions. Mediation analyses were conducted for biomarkers that have previously been demonstrated to have significant associations with breast cancer. Mediation analysis considered causal effects for changes in pollutant levels from the 25th to the 75th percentile and each mediator fixed at its median level. To test the robustness of our results, we further performed sensitivity mediation analyses, using average exposure from the inclusion to the date of blood collection. All multivariable models were adjusted for confounding factors identified by a direct acyclic graph (Supplementary Fig. 2), including body mass index, menopausal hormone replacement therapy use, urban/rural status at birth, urban/rural status at inclusion, alcohol drinking, breastfeeding, mammography before inclusion, oral contraceptive use, age at full-term pregnancy and parity, smoking status, total physical activity.

Analyses were conducted using R software version 4.2.3. Mediation analyses were conducted using STATA 14.

## Results

### Study population

Descriptive characteristics of the study population are shown in Supplementary Table 1, comprising 523 breast cancer cases and 523 matched controls. The mean age (± SD) at inclusion was 49.9 (± 6.3) years. Alcohol consumption was slightly lower in cases as compared to controls, with 52.0% of cases and 56.2% of controls reporting drinking more than 6.7 g/day. Education levels were generally high, with over 85% of participants having at least a 1- to 2-year university degree, with no difference between cases and controls. With the exception of breastfeeding, slightly more common in controls than in cases (62.5% vs. 59.1%), all other reproductive factors (age at first menstruation, use of oral contraceptives, and the number of children and age at first pregnancy) were overall similar between cases and controls. The distribution of body mass index, physical activity levels, and smoking status were also comparable between cases and controls.

Biomarker levels and annual mean concentration levels of pollutant exposure (NO_2_, BaP and PCB153) between cases and controls are shown in Supplementary Table 2 and Supplementary Table 3. There was no strong difference in the mean levels of all biomarkers between cases and controls. The average (±SD) of annual mean concentrations was 37.08 (±16.94) μg/m^3^, 0.21 (±0.12) ng/m^3^ and 11.06 (±4.04) ng/m^3^, for NO_2_, BaP and PCB153, respectively. The averages of annual mean concentrations of the three air pollutants were similar between cases and controls.

**Fig. 2:**
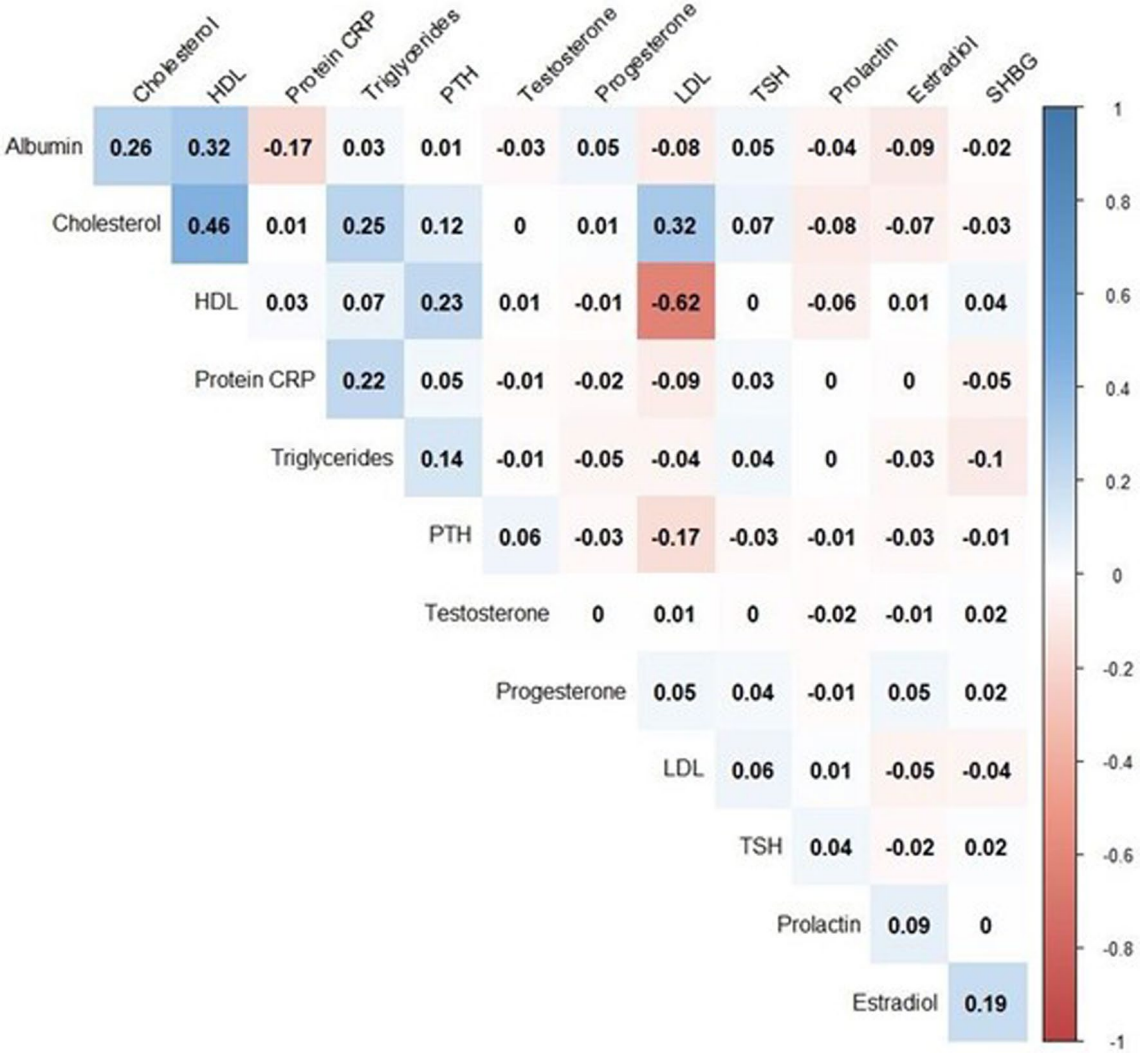
Pearson correlations between biomarkers. HDL: High-density lipoprotein cholesterol, LDL: Light-density lipoprotein cholesterol, TSH: Thyroid-stimulating hormone, SHGB: Sex Hormone-Binding Globulin, PTH: Parathormone, Protein CRP: C-reactive protein

Figure 2 presents Pearson correlation coefficients between biomarkers. Overall, with the exception of between HDL and LDL cholesterol (coef. = − 0.62), there were no strong correlations between biomarkers.

### Pollutant exposure and breast cancer risk

Table 1 presents the results of multivariable-adjusted associations between the three pollutants of interest and breast cancer risk. Overall, in continuous analyses, each SD increment in exposure to BaP (0.126 ng/m^3^) and NO_2_ (17.0 μg/m^3^) was associated with ORs of 1.04 (CI 0.81–1.34) and 1.04 (CI 0.81–1.34) of breast cancer risk, respectively. In contrast, exposure to PCB153 showed a borderline positive association, with an OR of 1.30 (CI 0.98–1.73) for each 1 SD increment in PCB153 levels (3.92 ng/m^3^).

**Table 1:** Associations between each pollutant and breast cancer risk

		NO_2_	BaP	PCB153
	Cases/Controls	OR (95% CI)	OR (95% CI)	OR (95% CI)
Continuous (For each 1SD increase)	523/523	1.04 (0.81, 1.34)	1.04 (0.81, 1.34)	1.30 (0.98, 1.73)
Quartiles				
I	121/131	1 (ref)	1 (ref)	1 (ref)
II	134/131	1.11 (0.74, 1.66)	1.58 (0.96, 2.61)	1.19 (0.68, 2.06)
III	132/130	1.14 (0.71, 1.83)	2.03 (1.05, 3.93)	2.25 (1.13, 4.57)
IV	136/131	1.18 (0.66, 2.11)	2.32 (1.00, 5.37)	2.07 (0.93, 4.61)

Conditional logistic regression models were used for estimating ORs and 95%CI, adjusted for body mass index, menopausal hormone replacement therapy uses, urban/rural status at birth, urban/rural status at inclusion, alcohol drinking, breastfeeding, mammography before inclusion, oral contraceptive use, age at full-term pregnancy and parity, smoking status, total physical activity.

The ORs (95% CI) correspond to an increment of 1 SD level in controls, NO_2_: 17.0 μg/m3, PCB153: 3.92 ng/m3, BaP: 0.126 ng/m^3^

Quartiles’ cut-offs for NO_2_ based on the distribution among controls: ≤ 24.2, ≤ 32.4, ≤ 46.5 µg/m^3^

Quartiles’ cut-offs for PCB153 based on the distribution among controls: ≤ 8.13, ≤ 10.12, ≤ 12.91 ng/m^3^

Quartiles’ cut-offs for BaP based on the distribution among controls: ≤ 0.133, ≤ 0.179, ≤ 0.240 ng/m^3^

SD Standard deviation, OR odds ratio, 95% CI 95% confidence intervals, NO_2_ nitrogen dioxide, BaP: benzo[a]pyrene, PCB153: polychlorinated biphenyls

In the analysis by quartiles, an increase in breast cancer risk was shown with increasing quartiles of BaP exposure (OR_Q3 vs Q1 _= 2.03, CI 1.05–3.93; and OR_Q4 vs Q1 _= 2.32, CI 1.00–5.37). Similarly, an increased risk of breast cancer associated with PCB153 exposure was observed for the third quartile (OR_Q3 vs Q1 _= 2.25, CI 1.13–4.57). However, the association became statistically non-significant in the highest exposed quartile (OR_Q4 vs Q1 _= 2.07, CI 0.93–4.61).

### Biomarkers and breast cancer risk

Table 2 shows the multivariable-adjusted ORs of the relationship between biomarkers of interest and breast cancer risk. Thyroid-stimulating hormone was inversely associated with breast cancer risk (OR = 0.87, CI 0.75–1.00, for each 1 SD increment), while estradiol was related to an increased risk of breast cancer (OR = 1.22, CI 1.05–1.42, for each 1 SD increment).

**Table 2:** Associations between each biomarker and breast cancer risk

Biomarkers	Cases/Controls	OR (CI 95%)	*P* value
Albumin	478 / 478	1.05 (0.90, 1.23)	0.53
Protein C-reactive	481 / 481	1.05 (0.90, 1.23)	0.52
Triglycerides	430 / 430	0.96 (0.83, 1.11)	0.56
Cholesterol	450 / 450	0.94 (0.82, 1.09)	0.41
HDL cholesterol	420 / 420	0.95 (0.76, 1.18)	0.62
LDL cholesterol	423 / 423	0.89 (0.72, 1.10)	0.29
Parathormone	470 / 470	0.92 (0.80, 1.06)	0.23
Thyroid-stimulating hormone	480 / 480	0.87 (0.75, 1.00)	0.04
Prolactin	484 / 484	1.01 (0.87, 1.16)	0.93
Estradiol	479 / 479	1.22 (1.05, 1.42)	0.01
Testosterone	472 / 472	1.03 (0.89, 1.20)	0.68
SHBG	440 / 440	0.97 (0.84, 1.11)	0.63
Progesterone	481 / 481	1.07 (0.93, 1.22)	0.35

Conditional logistic regression models were used for estimating ORs and 95%CI, for each 1SD biomarker increment, adjusted for body mass index, menopausal hormone replacement therapy uses, urban/rural status at birth, urban/rural status at inclusion, alcohol drinking, breastfeeding, mammography before inclusion, oral contraceptive use, age at full-term pregnancy and parity, smoking status, total physical activity.

OR: odds ratio; 95% CI: 95% confidence intervals, HDL cholesterol: High-density lipoprotein cholesterol, LDL cholesterol: Light-density lipoprotein cholesterol, SD: standard deviation, SHGB: Sex Hormone-binding globulin

*P* value was obtained based on Wald test.

### Pollutants and biomarkers associations

Results for the associations between pollutants (NO_2_, BaP and PCB153) and biomarkers are presented in Table 3. There was evidence of positive associations between albumin and each of the three pollutants. HDL cholesterol and LDL cholesterol were respectively, positively and inversely related to BaP. PTH was inversely associated with PCB153 and NO_2_. CRP and estradiol showed, respectively, inverse and positive associations with BaP.

**Table 3:** Beta coefficients and *P* values for associations between pollutants and biomarkers, a nested case–control study within the E3N-Generations cohort, 1990–2011

		BaP		PCB153		NO_2_	
Biomarkers	n	Beta	*P* value	Beta	*P* value	Beta	*P* value
Albumin	1001	0.088	0.007	0.094	0.003	0.071	0.048
Protein C-reactive	1003	− 0.061	0.057	− 0.034	0.264	− 0.045	0.181
Triglycerides	945	− 0.022	0.545	− 0.038	0.250	− 0.045	0.227
Cholesterol	972	0.009	0.806	− 0.011	0.751	− 0.017	0.649
HDL cholesterol	936	0.104	0.004	0.043	0.194	− 0.048	0.198
LDL cholesterol	937	− 0.092	0.012	− 0.060	0.079	0.039	0.305
Parathormone	992	− 0.053	0.131	− 0.116	0.001	− 0.155	0.000
TSH	1003	− 0.018	0.582	0.019	0.551	0.020	0.577
Prolactin	1007	0.035	0.282	0.045	0.154	0.043	0.223
Estradiol	1002	0.063	0.054	0.052	0.102	0.050	0.161
Testosterone	993	− 0.048	0.148	− 0.010	0.751	− 0.026	0.475
SHBG	960	0.036	0.291	− 0.006	0.848	− 0.004	0.920
Progesterone	1004	0.031	0.374	0.029	0.391	0.013	0.736

Linear regression models were used for estimating beta value, per 1 SD biomarker and pollutant increment,adjusted for body mass index, menopausal hormone replacement therapy uses, urban/rural status at birth, urban/rural status at inclusion, alcohol drinking, breastfeeding, and mammography before inclusion, oral contraceptive use, age at full-term pregnancy and parity, smoking status, total physical activity.

*P* value was obtained based on student test.

SD: standard deviation, HDL cholesterol: High-density lipoprotein cholesterol, LDL cholesterol: Light-density lipoprotein cholesterol, TSH: Thyroid-stimulating hormone, SHGB: Sex Hormone-Binding Globulin, NO_2_: nitrogen dioxide, BaP: benzo[a]pyrene, PCB153: polychlorinated biphenyls

### Four-way decomposition mediation analysis

Table 4 presents the results of the causal mediation analysis with the four-way decomposition (i.e. control direct effect (CDE), reference interaction (INT_ref_), mediated interaction (INT_med_), pure indirect effect (PIE)) of the effect of NO_2_ on breast cancer risk mediated individually by different biomarkers. This mediation analysis considered the causal effects of changes in pollutant levels from the 25th to the 75th percentile, with each mediator set at its median value. The CDEs (the effect in the absence of mediation or interaction) of NO_2_ on breast cancer risk were very high, ranging from 80.6 to 121.1%, when holding estradiol and testosterone at their median levels, respectively. The overall mediated effects (sum of PIE and mediation interaction) through estradiol and PTH were suggestively positive, at 18.8 and 13.6% respectively (Table 4).

**Table 4:** Four-way decomposition of each mediator of the associations between NO_2_ and breast cancer risk

Mediation	Effect	Estimate (CI 95%)	*P* value	Proportion	*P* value
Albumin	TE	0.1178 (− 0.2666, 0.5022)	0.548		
	CDE	0.1126 (− 0.2724, 0.4976)	0.566	95.6%	< 0.001
	INTref	0.0031 (− 0.0260, 0.0323)	0.833	2.7%	0.842
	INTmed	− 0.0014 (− 0.0136, 0.0109)	0.829	− 1.1%	0.838
	PIE	0.0034 (− 0.0098, 0.0167)	0.612	2.9%	0.695
	O_M			1.8%	0.766
CRP	TE	0.1206 (− 0.2608, 0.5020)	0.536		
	CDE	0.1335 (− 0.2459, 0.5130)	0.490	110.7%	< 0.001
	INTref	− 0.0127 (− 0.0708, 0.0455)	0.670	− 10.5%	0.738
	INTmed	0.0004 (− 0.0052, 0.0060)	0.882	0.4%	0.887
	PIE	− 0.0007 (− 0.0099, 0.0084)	0.877	− 0.6%	0.881
	O_M			− 0.2%	0.893
Triglycerides	TE	0.1260 (− 0.2776, 0.5297)	0.541		
	CDE	0.1254 (− 0.2815, 0.5324)	0.546	99.5%	< 0.001
	INTref	− 0.0026 (− 0.0339, 0.0287)	0.873	− 2.0%	0.873
	INTmed	0.0009 (− 0.0104, 0.0122)	0.879	0.7%	0.879
	PIE	0.0023 (− 0.0099, 0.0145)	0.713	1.8%	0.749
	O_M			2.5%	0.681
Cholesterol	TE	0.0836 (− 0.2955, 0.4628)	0.665		
	CDE	0.0828 (− 0.2969, 0.4624)	0.669	99.0%	< 0.001
	INTref	− 0.0011 (− 0.0104, 0.0083)	0.824	− 1.3%	0.835
	INTmed	0.0012 (− 0.0066, 0.0090)	0.768	1.4%	0.796
	PIE	0.0008 (− 0.0051, 0.0066)	0.802	0.9%	0.829
	O_M			2.3%	0.787
HDL cholesterol	TE	0.1667 (− 0.2575, 0.5908)	0.441		
	CDE	0.1692 (− 0.2587, 0.5971)	0.438	101.5%	< 0.001
	INTref	− 0.0073 (− 0.0437, 0.0291)	0.695	− 4.4%	0.715
	INTmed	0.0030 (− 0.0134, 0.0195)	0.720	1.8%	0.736
	PIE	0.0017 (− 0.0145, 0.0179)	0.834	1.0%	0.840
	O_M			2.8%	0.680
LDL cholesterol	TE	0.2178 (− 0.2414, 0.6770)	0.353		
	CDE	0.2164 (− 0.2313, 0.6641)	0.343	99.4%	< 0.001
	INTref	0.0150 (− 0.0381, 0.0681)	0.579	6.9%	0.552
	INTmed	− 0.0073 (− 0.0325, 0.0180)	0.573	− 3.3%	0.554
	PIE	− 0.0064 (− 0.0278, 0.0151)	0.561	− 2.9%	0.629
	O_M			− 6.2%	0.495
Parathormone	TE	0.1378 (− 0.2679, 0.5436)	0.506		
	CDE	0.1247 (− 0.2761, 0.5254)	0.542	90.5%	< 0.001
	INTref	− 0.0055 (− 0.0234, 0.0123)	0.544	− 4.0%	0.626
	INTmed	0.0103 (− 0.0195, 0.0402)	0.497	7.5%	0.600
	PIE	0.0083 (− 0.0179, 0.0346)	0.533	6.1%	0.622
	O_M			13.6%	0.510
TSH	TE	0.0289 (− 0.3257, 0.3835)	0.873		
	CDE	0.0310 (− 0.3270, 0.3889)	0.865	107.3%	0.168
	INTref	− 0.0004 (− 0.0295, 0.0286)	0.978	− 1.4%	0.978
	INTmed	0.0033 (− 0.0176, 0.0241)	0.760	11.3%	0.893
	PIE	− 0.0050 (− 0.0363, 0.0264)	0.757	− 17.1%	0.890
	O_M			− 5.9%	0.888
Prolactin	TE	0.1544 (− 0.2442, 0.5531)	0.448		
	CDE	0.1568 (− 0.2435, 0.5570)	0.443	101.5%	< 0.001
	INTref	0.0025 (− 0.0148, 0.0198)	0.777	1.6%	0.786
	INTmed	− 0.0115 (− 0.0381, 0.0150)	0.394	− 7.5%	0.537
	PIE	0.0067 (− 0.0153, 0.0288)	0.549	4.4%	0.624
	O_M			− 3.1%	0.712
Estradiol	TE	0.0935 (− 0.2865, 0.4734)	0.630		
	CDE	0.0776 (− 0.3007, 0.4559)	0.688	83.0%	0.027
	INTref	− 0.0017 (− 0.0124, 0.0089)	0.751	− 1.8%	0.700
	INTmed	− 0.0035 (− 0.0232, 0.0162)	0.730	− 3.7%	0.807
	PIE	0.0210 (− 0.0133, 0.0554)	0.230	22.5%	0.647
	O_M			18.8%	0.619
Testosterone	TE	0.1167 (− 0.2850, 0.5184)	0.569		
	CDE	0.1414 (− 0.2610, 0.5437)	0.491	121.1%	0.004
	INTref	− 0.0195 (− 0.0708, 0.0317)	0.455	− 16.7%	0.666
	INTmed	− 0.0119 (− 0.0495, 0.0258)	0.536	− 10.2%	0.659
	PIE	0.0068 (− 0.0153, 0.0289)	0.548	5.8%	0.680
	O_M			− 4.4%	0.659
SHBG	TE	0.1452 (− 0.2664, 0.5569)	0.489		
	CDE	0.1446 (− 0.2669, 0.5561)	0.491	99.6%	< 0.001
	INTref	0.0005 (− 0.0082, 0.0092)	0.912	0.3%	0.912
	INTmed	− 0.0005 (− 0.0069, 0.0058)	0.866	− 0.4%	0.869
	PIE	0.0007 (− 0.0069, 0.0082)	0.860	0.5%	0.864
	O_M			0.1%	0.926
Progesterone	TE	0.1063 (− 0.2766, 0.4891)	0.586		
	CDE	0.0856 (− 0.2986, 0.4699)	0.662	80.6%	0.103
	INTref	0.0226 (− 0.0569, 0.1021)	0.578	21.3%	0.684
	INTmed	− 0.0011 (− 0.0094, 0.0072)	0.788	− 1.1%	0.808
	PIE	− 0.0008 (− 0.0071, 0.0055)	0.801	− 0.8%	0.818
	O_M			− 1.8%	0.794

TE: total effect (total excess relative risk), CDE: excess relative risk due to controlled direct effect, INTref: excess relative risk due to reference interaction, INTmed: excess relative risk due to mediated interaction, PIE: excess relative risk due to pure indirect effect, O_M: overall mediated

CRP: C-reactive protein, HDL: High-density lipoprotein cholesterol, LDL: Light-density lipoprotein cholesterol, SHBG: Sex Hormone-Binding Globulin, TSH: Thyroid-stimulating hormone, SHGB: Sex Hormone-binding globulin

Output of mediation analysis with causal effects estimated for a change in pollutant levels from the 25th to the 75th percentile

Adjusted for body mass index, menopausal hormone replacement therapy uses, urban/rural status at birth, urban/rural status at inclusion, alcohol drinking, breastfeeding, mammography before inclusion, oral contraceptive use, age at full-term pregnancy and parity, smoking status, total physical activity

Controlled direct effects are computed fixing the mediators at their median levels

For PCB153, the proportions of CDE were elevated, ranging from 95.2 to 106.0%, when holding estradiol and CRP at their median levels, respectively (Table 5). Although not statistically significant, small proportions of the association between PCB153 and breast cancer were mediated by estradiol and PTH, with the overall mediated effect being 6.4 and 4.1%, respectively (Table 5).

**Table 5:** Four-way decomposition of each mediator of the associations between PCB153 and breast cancer risk

Mediation	Effect	Estimate (CI 95%)	*P* value	Proportion	*P* value
Albumin	TE	0.4563 (− 0.0831, 0.9957)	0.097		
	CDE	0.4369 (− 0.1007, 0.9746)	0.111	95.7%	< 0.001
	INTref	0.0204 (− 0.0320, 0.0728)	0.445	4.5%	0.475
	INTmed	− 0.0139 (− 0.0443, 0.0164)	0.368	− 3.1%	0.413
	PIE	0.0129 (− 0.0119, 0.0377)	0.306	2.8%	0.375
	O_M			− 0.2%	0.946
CRP	TE	0.4211 (− 0.1049, 0.9471)	0.117		
	CDE	0.4462 (− 0.0882, 0.9806)	0.102	106.0%	< 0.001
	INTref	− 0.0251 (− 0.0821, 0.0319)	0.388	− 6.0%	0.435
	INTmed	− 0.0021 (− 0.0131, 0.0090)	0.711	− 0.5%	0.715
	PIE	0.0021 (− 0.0088, 0.0130)	0.705	0.5%	0.714
	O_M			0.0%	0.994
Triglycerides	TE	0.4763 (− 0.0883, 1.0410)	0.098		
	CDE	0.4948 (− 0.0772, 1.0667)	0.090	103.9%	< 0.001
	INTref	− 0.0184 (− 0.0507, 0.0139)	0.265	− 3.9%	0.305
	INTmed	− 0.0001 (− 0.0151, 0.0150)	0.997	0.0%	0.997
	PIE	0.0001 (− 0.0018, 0.0018)	0.997	0.0%	0.997
	O_M			0.0%	0.997
Cholesterol	TE	0.5117 (− 0.0634, 1.0868)	0.081		
	CDE	0.5158 (− 0.0611, 1.0928)	0.080	100.8%	< 0.001
	INTref	− 0.0017 (− 0.0232, 0.0197)	0.875	− 0.3%	0.875
	INTmed	− 0.0027 (− 0.0184, 0.0129)	0.730	− 0.5%	0.732
	PIE	0.0003 (− 0.0039, 0.0046)	0.873	0.1%	0.873
	O_M			− 0.5%	0.739
HDL cholesterol	TE	0.5204 (− 0.0624, 1.1032)	0.080		
	CDE	0.5379 (− 0.0546, 1.1303)	0.075	103.4%	< 0.001
	INTref	− 0.0118 (− 0.0427, 0.0191)	0.455	− 2.3%	0.455
	INTmed	− 0.0066 (− 0.0268, 0.0136)	0.523	− 1.3%	0.526
	PIE	0.0009 (− 0.0128, 0.0147)	0.894	0.2%	0.894
	O_M			− 1.1%	0.624
LDL cholesterol	TE	0.5723 (− 0.0436, 1.1882)	0.069		
	CDE	0.5481 (− 0.0487, 1.1449)	0.072	95.8%	< 0.001
	INTref	0.0121 (− 0.0355, 0.0597)	0.618	2.1%	0.604
	INTmed	0.0079 (− 0.0177, 0.0335)	0.545	1.4%	0.525
	PIE	0.0042 (− 0.0117, 0.0200)	0.605	0.7%	0.611
	O_M			2.1%	0.497
Parathormone	TE	0.3785 (− 0.1522, 0.9092)	0.162		
	CDE	0.3702 (− 0.1577, 0.8982)	0.169	97.8%	< 0.001
	INTref	− 0.0073 (− 0.0255, 0.0109)	0.430	− 1.9%	0.456
	INTmed	0.0109 (− 0.0123, 0.0341)	0.357	2.9%	0.393
	PIE	0.0047 (− 0.0144, 0.0237)	0.631	1.2%	0.638
	O_M			4.1%	0.332
TSH	TE	0.3490 (− 0.1430, 0.8410)	0.164		
	CDE	0.3608 (− 0.1374, 0.8591)	0.156	103.4%	< 0.001
	INTref	− 0.0071 (− 0.0309, 0.0167)	0.559	− 2.0%	0.565
	INTmed	0.0027 (− 0.0091, 0.0145)	0.654	0.8%	0.689
	PIE	− 0.0075 (− 0.0354, 0.0205)	0.600	− 2.1%	0.631
	O_M			− 1.4%	0.631
Prolactin	TE	0.4295 (− 0.0944, 0.9534)	0.108		
	CDE	0.4299 (− 0.0946, 0.9543)	0.108	100.1%	< 0.001
	INTref	0.0003 (− 0.0051, 0.0058)	0.905	0.1%	0.905
	INTmed	− 0.0022 (− 0.0181, 0.0136)	0.782	− 0.5%	0.784
	PIE	0.0015 (− 0.0133, 0.0163)	0.841	0.4%	0.842
	O_M			− 0.2%	0.940
Estradiol	TE	0.3752 (− 0.1330, 0.8835)	0.148		
	CDE	0.3570 (− 0.1500, 0.8641)	0.168	95.2%	< 0.001
	INTref	− 0.0060 (− 0.0195, 0.0074)	0.381	− 1.6%	0.248
	INTmed	0.0079 (− 0.0160, 0.0318)	0.517	2.1%	0.474
	PIE	0.0163 (− 0.0102, 0.0427)	0.227	4.3%	0.345
	O_M			6.4%	0.266
Testosterone	TE	0.3552 (− 0.1657, 0.8762)	0.181		
	CDE	0.3759 (− 0.1457, 0.8975)	0.158	105.8%	< 0.001
	INTref	− 0.0175 (− 0.0597, 0.0247)	0.416	− 4.9%	0.502
	INTmed	− 0.0075 (− 0.0344, 0.0194)	0.584	− 2.1%	0.601
	PIE	0.0044 (− 0.0118, 0.0206)	0.595	1.2%	0.624
	O_M			− 0.9%	0.649
SHBG	TE	0.5035 (− 0.0961, 1.1030)	0.100		
	CDE	0.5021 (− 0.0975, 1.1016)	0.101	99.7%	< 0.001
	INTref	0.0015 (− 0.0180, 0.0211)	0.878	0.3%	0.879
	INTmed	− 0.0034 (− 0.0168, 0.0101)	0.624	− 0.7%	0.636
	PIE	0.0032 (− 0.0089, 0.0154)	0.600	0.6%	0.614
	O_M			0.0%	0.979
Progesterone	TE	0.4212 (− 0.0995, 0.9420)	0.113		
	CDE	0.4321 (− 0.0897, 0.9539)	0.105	102.6%	< 0.001
	INTref	− 0.0138 (− 0.0936, 0.0661)	0.735	− 3.3%	0.744
	INTmed	− 0.0015 (− 0.0115, 0.0086)	0.772	− 0.4%	0.778
	PIE	0.0044 (− 0.0095, 0.0183)	0.537	1.0%	0.561
	O_M			0.7%	0.646

TE: total effect (total excess relative risk), CDE: excess relative risk due to controlled direct effect, INTref: excess relative risk due to reference interaction, INTmed: excess relative risk due to mediated interaction, PIE: excess relative risk due to pure indirect effect, O_M: overall mediated

CRP: C-reactive protein, HDL: High-density lipoprotein cholesterol, LDL: Light-density lipoprotein cholesterol, SHBG: Sex Hormone-Binding Globulin, TSH: Thyroid-stimulating hormone, SHGB: Sex Hormone-binding globulin

Output of mediation analysis with causal effects estimated for a change in pollutant levels from the 25th to the 75th percentile

Adjusted for body mass index, menopausal hormone replacement therapy uses, urban/rural status at birth, urban/rural status at inclusion, alcohol drinking, breastfeeding, mammography before inclusion, oral contraceptive use, age at full-term pregnancy and parity, smoking status, total physical activity

Controlled direct effects are computed fixing the mediators at their median levels

Table 6 displays the results of the causal mediation analysis with four-way decompositions of the effect of BaP on breast cancer mediated individually by different biomarkers. The CDEs ranged from 66.4 to 176.5% while holding albumin and progesterone at their median levels, respectively. The overall mediated effects through albumin (24.3%), LDL cholesterol (22.8%), and estradiol (27.0%) were suggestively positive. In contrast, there was a non-significant negative mediated effect through HDL cholesterol (− 18.7%).

**Table 6:** Four-way decomposition of each mediator of the associations between BaP and breast cancer risk

Mediation	Effect	Estimate (CI 95%)	*P* value	Proportion	*P* value
Albumin	TE	0.0143 (− 0.2134, 0.2421)	0.902		
	CDE	0.0095 (− 0.2189, 0.2380)	0.935	66.4%	0.813
	INTref	0.0013 (− 0.0175, 0.0201)	0.890	9.3%	0.928
	INTmed	− 0.0007 (− 0.0101, 0.0088)	0.889	− 4.7%	0.927
	PIE	0.0042 (− 0.0096, 0.0179)	0.553	29.0%	0.903
	O_M			24.3%	0.903
CRP	TE	0.0530 (− 0.2032, 0.3091)	0.685		
	CDE	0.0705 (− 0.1792, 0.3201)	0.580	133.0%	0.216
	INTref	− 0.0160 (− 0.0737, 0.0418)	0.587	− 30.2%	0.769
	INTmed	0.0019 (− 0.0057, 0.0094)	0.629	3.5%	0.779
	PIE	− 0.0034 (− 0.0132, 0.0064)	0.501	− 6.3%	0.736
	O_M			− 2.8%	0.762
Triglycerides	TE	0.0287 (− 0.2204, 0.2778)	0.821		
	CDE	0.0255 (− 0.2236, 0.2746)	0.841	88.9%	0.127
	INTref	0.0027 (− 0.0161, 0.0215)	0.778	9.4%	0.858
	INTmed	− 0.0002 (− 0.0027, 0.0022)	0.839	− 0.9%	0.877
	PIE	0.0007 (− 0.0046, 0.0061)	0.789	2.5%	0.861
	O_M			1.7%	0.865
Cholesterol	TE	0.0935 (− 0.1848, 0.3717)	0.510		
	CDE	0.0973 (− 0.1822, 0.3768)	0.495	104.0%	< 0.001
	INTref	0.0006 (− 0.0191, 0.0204)	0.949	0.7%	0.949
	INTmed	− 0.0056 (− 0.0172, 0.0059)	0.339	− 6.0%	0.547
	PIE	0.0012 (− 0.0074, 0.0099)	0.783	1.3%	0.793
	O_M			− 4.7%	0.580
HDL cholesterol	TE	0.0977 (− 0.2007, 0.3962)	0.521		
	CDE	0.1207 (− 0.1885, 0.4299)	0.444	123.5%	< 0.001
	INTref	− 0.0047 (− 0.0451, 0.0357)	0.821	− 4.8%	0.828
	INTmed	− 0.0262 (− 0.0599, 0.0076)	0.129	− 26.8%	0.487
	PIE	0.0078 (− 0.0287, 0.0443)	0.674	8.0%	0.705
	O_M			− 18.7%	0.562
LDL cholesterol	TE	0.0560 (− 0.2016, 0.3135)	0.670		
	CDE	0.0436 (− 0.2102, 0.2973)	0.736	77.9%	0.147
	INTref	− 0.0004 (− 0.0079, 0.0071)	0.920	− 0.7%	0.920
	INTmed	− 0.0003 (− 0.0200, 0.0195)	0.979	− 0.5%	0.979
	PIE	0.0130 (− 0.0149, 0.0410)	0.361	23.3%	0.683
	O_M			22.8%	0.670
Parathormone	TE	0.0337 (− 0.2019, 0.2693)	0.779		
	CDE	0.0324 (− 0.2020, 0.2668)	0.786	96.1%	0.002
	INTref	− 0.0020 (− 0.0186, 0.0147)	0.815	− 5.9%	0.860
	INTmed	0.0026 (− 0.0055, 0.0107)	0.532	7.7%	0.790
	PIE	0.0007 (− 0.0043, 0.0056)	0.783	2.1%	0.837
	O_M			9.7%	0.787
TSH	TE	0.0376 (− 0.1991, 0.2742)	0.756		
	CDE	0.0342 (− 0.2051, 0.2735)	0.779	91.1%	0.012
	INTref	0.0004 (− 0.0066, 0.0075)	0.904	1.2%	0.922
	INTmed	− 0.0006 (− 0.0041, 0.0030)	0.761	− 1.5%	0.839
	PIE	0.0035 (− 0.0097, 0.0166)	0.604	9.3%	0.785
	O_M			7.8%	0.779
Prolactin	TE	0.0369 (− 0.1988, 0.2727)	0.759		
	CDE	0.0348 (− 0.1997, 0.2693)	0.771	94.2%	< 0.001
	INTref	0.0012 (− 0.0114, 0.0138)	0.854	3.2%	0.869
	INTmed	0.0020 (− 0.0046, 0.0086)	0.548	5.5%	0.780
	PIE	− 0.0011 (− 0.0062, 0.0041)	0.685	− 2.9%	0.810
	O_M			2.6%	0.799
Estradiol	TE	0.0434 (− 0.1963, 0.2831)	0.722		
	CDE	0.0321 (− 0.2067, 0.2709)	0.792	74.0%	0.320
	INTref	− 0.0004 (− 0.0043, 0.0035)	0.837	− 0.9%	0.765
	INTmed	0.0005 (− 0.0077, 0.0086)	0.910	1.1%	0.899
	PIE	0.0112 (− 0.0062, 0.0287)	0.208	25.9%	0.731
	O_M			27.0%	0.714
Testosterone	TE	0.0249 (− 0.2116, 0.2613)	0.837		
	CDE	0.0251 (− 0.2073, 0.2576)	0.832	101.1%	0.008
	INTref	0.0006 (− 0.0187, 0.0200)	0.948	2.6%	0.947
	INTmed	− 0.0001 (− 0.0043, 0.0040)	0.948	− 0.6%	0.946
	PIE	− 0.0008 (− 0.0058, 0.0043)	0.765	− 3.1%	0.872
	O_M			− 3.6%	0.853
SHBG	TE	0.0810 (− 0.1913, 0.3533)	0.560		
	CDE	0.0807 (− 0.1918, 0.3533)	0.561	99.7%	< 0.001
	INTref	0.0001 (− 0.0097, 0.0099)	0.986	0.1%	0.986
	INTmed	− 0.0006 (− 0.0053, 0.0040)	0.784	− 0.8%	0.802
	PIE	0.0008 (− 0.0050, 0.0066)	0.783	1.0%	0.801
	O_M			0.2%	0.879
Progesterone	TE	0.0326 (− 0.2022, 0.2674)	0.785		
	CDE	0.0576 (− 0.1825, 0.2976)	0.638	176.5%	0.550
	INTref	− 0.0271 (− 0.0789, 0.0247)	0.305	− 83.1%	0.794
	INTmed	− 0.0038 (− 0.0125, 0.0050)	0.399	− 11.5%	0.795
	PIE	0.0059 (− 0.0061, 0.0179)	0.335	18.1%	0.794
	O_M			6.5%	0.813

TE: total effect (total excess relative risk), CDE: excess relative risk due to controlled direct effect, INTref: excess relative risk due to reference interaction, INTmed: excess relative risk due to mediated interaction, PIE: excess relative risk due to pure indirect effect, O_M: overall mediated

CRP: C-reactive protein, HDL: High-density lipoprotein cholesterol, LDL: Light-density lipoprotein cholesterol, SHBG: Sex Hormone-Binding globulin, TSH: Thyroid-stimulating hormone, SHGB: Sex Hormone-binding globulin

Output of mediation analysis with causal effects estimated for a change in pollutant levels from the 25th to the 75th percentile

Adjusted for body mass index, menopausal hormone replacement therapy uses, urban/rural status at birth, urban/rural status at inclusion, alcohol drinking, breastfeeding, mammography before inclusion, oral contraceptive use, age at full-term pregnancy and parity, smoking status, total physical activity

Controlled direct effects are computed fixing the mediators at their median levels

The sensitivity mediation analyses, which restricted pollutants exposure to the period from inclusion to the date of biomarker collection, yielded comparable mediating effects to those observed when exposure was measured until the index date (Supplementary Tables 4, 5 and 6).

## Discussion

This study is, to date, the first to assess whether specific biomarkers act as potential mediators of the association between exposure to three major air pollutants (NO_2_, BaP and PCB153) and risk of breast cancer. Our analyses revealed a significantly increased risk of breast cancer with increasing quartile levels of BaP and PCB153 exposures. A positive but not statistically significant association was observed between exposure to NO_2_ and the risk of breast cancer. There was evidence of an inverse association between thyroid-stimulating hormone and breast cancer risk, whereas estradiol showed an increased risk of breast cancer. The four-way decomposition mediation analysis showed a suggestive mediation through estradiol and PTH in the association of NO_2_ and PCB153 exposures with breast cancer risk, whereas albumin, estradiol, LDL and HDL cholesterol may play a role in the association between BaP and breast cancer risk.

BaP and PCB153 are recognized as EDP [7]. Steroid hormones, especially estradiol, have been strongly linked to the risk of breast cancer [48, 49]. Certain EDP may promote tumor growth through pathways mediated by estrogen, progesterone, or other hormonal responses, particularly by modifying the levels of these steroid hormones [50, 51]. Moreover, PAHs, such as BaP, exhibit estrogenic properties and could, therefore, stimulate the proliferation of breast cells [23]. Certain BaP metabolites can bind to estrogen receptors and activate estrogen-dependent signalling pathways, potentially promoting the growth of breast cells [52]. Although a direct link between NO_2_ and estradiol has not been established, NO_2_ can contribute to both endocrine disruption (ED) and carcinogenic effects through indirect mechanisms [53–55]. Notably, its potential carcinogenicity can arise from pathways such as oxidative stress and chronic inflammation, influencing various cancer-related processes (such as angiogenesis, apoptosis, cell cycle regulation, invasion, and metastasis) or enhancing the effects of other environmental carcinogens. Overall, these findings are in agreement with our mediation results observed for estradiol, suggesting a potential role in the association between these pollutants and breast cancer development.

Furthermore, we noted an elevated but statistically non-significant mediated proportion for PTH in the association between both NO_2_ and PCB153 exposures and breast cancer risk. PTH is a peptide hormone secreted by the parathyroid glands, playing a crucial role in the metabolism of calcium and phosphorus [56]. A few studies have suggested that PTH might be involved in the development of breast cancer [57, 58]. Although the association between PTH and PCB153 or NO_2_ has not yet been well studied, some studies have found associations between PTH and other air pollutants [59, 60]. Specifically, an inverse association was observed between particulate matter <2.5 μm diameter (PM_2.5_) exposure and PTH levels [59]. Another study provides insights into the impact of ozone (O_3_) on PTH levels [60].

While we observed a higher positive indirect effect through LDL cholesterol (21%), there was a suggestive negative through HDL cholesterol (− 17%) in the association between BaP exposure and breast cancer. This negative effect is mainly due to the opposite associations between exposure-biomarker and biomarker-outcome, indicating antagonistic associations between the effect of BaP on HDL cholesterol (positive association) and the association of HDL cholesterol with breast cancer risk (inverse association), resulting in an overall negative mediated proportion. Several studies have identified associations between high levels of LDL cholesterol or low levels of HDL cholesterol and an increased risk of breast cancer [61–63]. Furthermore, studies have demonstrated a link between exposure to certain EDP, such as bisphenol A or perfluorinated compounds and cholesterol [64, 65]. However, a direct link between BaP and cholesterol has not been investigated in previous studies.

In contrast, we estimated an important mediation through albumin (i.e., proportion of pure indirect effect = 28%) for the association between BaP and risk of breast cancer. Regarding the role of albumin, its levels have been reported to be associated with breast cancer risk [66]. The mediating effect of albumin in the association between BaP and breast cancer development has not been investigated in other studies yet.

Although we did not identify potential mediating effects of metabolic/inflammatory markers, previous studies reported that chronic inflammatory and metabolism conditions play a role in the underlying mechanisms linking air pollution and breast cancer risk [54, 66]. Both BaP and PCB exposures can result in perturbation of inflammation mediators, leading to an inflammation microenvironment (via TNF-α and NFκB leading to IL-6 upregulation) that facilitates and contributes to the migration and invasion of breast cancer cells [67, 68]. Taken together, all these conditions can stimulate the growth of breast cancer cells and contribute to the development and progression of breast cancer.

Our study has several strengths. One main strength is the use of four-way decomposition mediation analyses to explore potential mediating pathways linking air pollutants to the risk of breast cancer. The method used in this study has several advantages compared to other mediation analysis approaches, including the ability to estimate the reference interaction and mediated interaction, greater flexibility and better control of confounding variables. In addition, this study has investigated several biomarkers of metabolic health, adjusted all the models for a comprehensive list of confounding variables. While this present study is the first to explore the potential mediation role of several biomarkers of metabolic health, the findings offer insights into the potential biological pathways through which these pollutants could influence the risk of breast cancer development, and suggest promising research perspectives. In the present study, biases due to exposure occurring after biomarker assessment are unlikely, as our additional sensitivity mediation analyses using the average exposure from the time of inclusion to the date of biomarker assessment, revealed no substantial differences as compared to the exposure calculated from inclusion to the index date. These findings confirm the robustness of the estimates and suggest that the timing of exposure relative to biomarker collection did non influence the results observed in our mediation analyses. However, further studies with larger sample sizes are needed to confirm and extend these findings. A better understanding of underlying mechanisms could lead to more effective preventive strategies for breast cancer.

A notable limitation of the present study is the limited statistical power due to small sample size, which may reduce our ability to detect significant associations, especially in mediation analyses. Additionally, the small sample size precluded us from performing stratified analyses. Despite the extensive efforts to adjust for a potential confounder, residual confounding cannot be entirely excluded. We noted some negative proportions and proportions exceeding 100%. As mentioned earlier, negative effects can occur when the associations between exposure-biomarker and biomarker-outcome are in the opposite direction, leading to proportions of the overall effect exceeding 100%. However, in some cases, these negative effects may be attributable to confounding or interaction with other variables, or measurement biases. It should be noted that due to sample size limitations, we were not able to perform multiple-mediator models; future studies, with larger sample sizes should consider the simultaneous analysis of multiple mediators, which could provide insights into how each biomarker contributes to the overall mediated effect. Additionally, limitations associated with multiple-mediator models, such as collinearity, should be carefully managed to ensure robust findings. It is also important to note the lack of representativeness in the study sample, since the analysis was based on a subsample of the E3N cohort participants, who were predominantly teachers. Thus, caution is warranted in interpreting these results or extrapolating them to the general population. Finally, the results should also be interpreted with caution due to the wide confidence intervals, which may indicate a degree of uncertainty and precision in the estimates.

## Conclusion

Overall, this pioneering study provides additional insights into the potential role of several metabolic health biomarkers in mediating the association between air pollutants and breast cancer risk. Although not statistically significant, there was a suggestive mediation through estradiol and PTH in the association of NO_2_ and PCB153 exposures with breast cancer risk. Similarly, albumin, estradiol, and both LDL and HDL cholesterol may play a role in linking BaP exposure to breast cancer risk. These findings emphasize the need and importance of further investigation into the role of biomarkers linking air pollutant exposure to the occurrence of breast cancer, a major public health issue. This study also highlights the value of mediation analysis in unravelling the complex mechanisms through which environmental exposures may impact global human health.

## Supplementary Information

Additional file 1

## Data Availability

The datasets used and/or analysed during the current study are available from the corresponding author on reasonable request.

## Abbreviations

BaP: Benzo[a]pyrene
BC: Breast cancer
BCG: Bromocresol green
CI: Confidence intervals
CDE: Controlled direct effect
CNIL: National Commission for Data Protection and Privacy
CRP: C-reactive protein
E3N: Etude Epidémiologique auprès de femmes de la Mutuelle Générale de l’Education Nationale
ECLIA: Electrochemiluminescence immunoassay
ED: Endocrine-disrupting
HDL: High-density lipoproteins cholesterol
IARC: International Agency for Research on Cancer
INTmed: Mediated interaction effect
INTref: Reference interaction effect
LDL: Low-density lipoproteins cholesterol
LUR: Land use regression
NO_2_: Nitrogen dioxide
ORs: Odds ratios
O_3_: Ozone
PAHs: Polycyclic aromatic hydrocarbons
PCBs: Polychlorinated biphenyls
PCB153: Group III of the Wolff’s classification of polychlorinated biphenyls
PTH: Parathormone
PIE: Pure Indirect Effect
SHBG: Sex hormone-binding globulin
SD: Standard deviation
TE: Total effect
TRAP: Traffic-related air pollutants
