# Supplementary material for: Exposure to air pollutants and breast cancer risk: mediating effects of metabolic health biomarkers in a nested case–control study within the E3N-Generations cohort
Source: Breast Cancer Res. 2024 Nov 15;26:159. doi: 10.1186/s13058-024-01913-7 (PMC11568591; doi:10.1186/s13058-024-01913-7)
Supplement: Supplementary file 1 — Additional file 1 [file 13058_2024_1913_MOESM1_ESM.docx]

**Supplementary material**

**Exposure to air pollutants and breast cancer risk: mediating effects of metabolic health biomarkers in a nested case-control study within the E3N-Generations cohort**

Benoît Mercoeur^a,b^, Béatrice Fervers^a,b^, Delphine Praud^a,b^, Hwayoung Noh^a,b^, Thomas Coudon^a,b^, Camille Giampiccolo^a,b,c,d^, Lény Grassot^a,b^, Elodie Faure^e^, Florian Couvidat^f^, Gianluca Severi^e,g^, Francesca Romana Mancini^e^, Pascal Roy^c,d,h^, and Amina Amadou^a,b^

Affiliations :

^a^ Department of Prevention Cancer Environnement, Centre Léon Bérard, Lyon, France

^b^ Inserm U1296 Radiations : Défense, Santé, Environnement, Lyon, France

^c^ Laboratoire de Biométrie Et Biologie Evolutive, CNRS UMR 5558, Villeurbanne, France

^d^ Service de Biostatistique-Bioinformatique, Pole Sante Publique, Hospices Civils de Lyon, Lyon, France

^e^ Universite Paris-Saclay, UVSQ, Inserm, Gustave Roussy, CESP, 94805 Villejuif, France

^f^ National Institute for industrial Environment and Risks (INERIS), Verneuil-en-Halatte, France

^g^ Department of Statistics, Computer Science and Applications (DISIA), University of Florence, Florence, Italy

^h^ Université Claude Bernard Lyon 1, Lyon, France

**Table of contents**

Fig. S1: Flowchart of the selection of the study participants

Fig. S2: Directed Acyclic Graph showing the relationship between potential confounders in the association between air pollutants and breast cancer risk

Table S1: Demographic and lifestyle characteristics of breast cancer cases and matched controls in a nested case-control study within the French E3N-Generations cohort,1990-2011

Table S2: Biomarker levels of breast cancer cases and matched controls in a nested case-control study within the French E3N-Generations cohort, 1990-2011

Table S3: Pollutant levels exposition of breast cancer cases and matched controls in a nested case-control study within the French E3N-Generations cohort, 1990-2011

Table S4: Beta coefficient (CI95%) and proportion attributable for the four-way decomposition of each mediator of the associations between NO_2_ exposure from inclusion to biomarker collection date and breast cancer risk, a nested case-control study within the E3N-Generations cohort, 1990-2011

Table S5: Beta coefficient (CI95%) and proportion attributable for the four-way decomposition of each mediator of the associations between PCB153 exposure from inclusion to biomarker collection date and breast cancer risk, a nested case-control study within the E3N-Generations cohort, 1990-2011

Table S6: Beta coefficient (CI95%) and proportion attributable for the four-way decomposition of each mediator of the associations between BaP exposure from inclusion to biomarker collection date and breast cancer risk, a nested case-control study within the E3N-Generations cohort, 1990-2011

**E3N Cohort**

N=98,995

**Matched Controls**

N=6,298

**Breast cancers cases**

N=6,298

**Paget disease, phyllodes tumors and their matched controls**

N=19 + 19

**Subjects with missing data on matching variables, and their matched subjects**

N= 3 + 3

**Subjects with missing address or outside the continental mainland France, and their matched subjects**

N=1,054 + 1,054

**Xenair study**

N=5222 + 5222

**Subjects without biomarker measurements, and their matched subjects**

N=4,687 + 4,687

**Matched Controls**

N=523

**Breast cancers cases**

N=523

**Supplementary Figure 1:** Flowchart of study participant selection

**
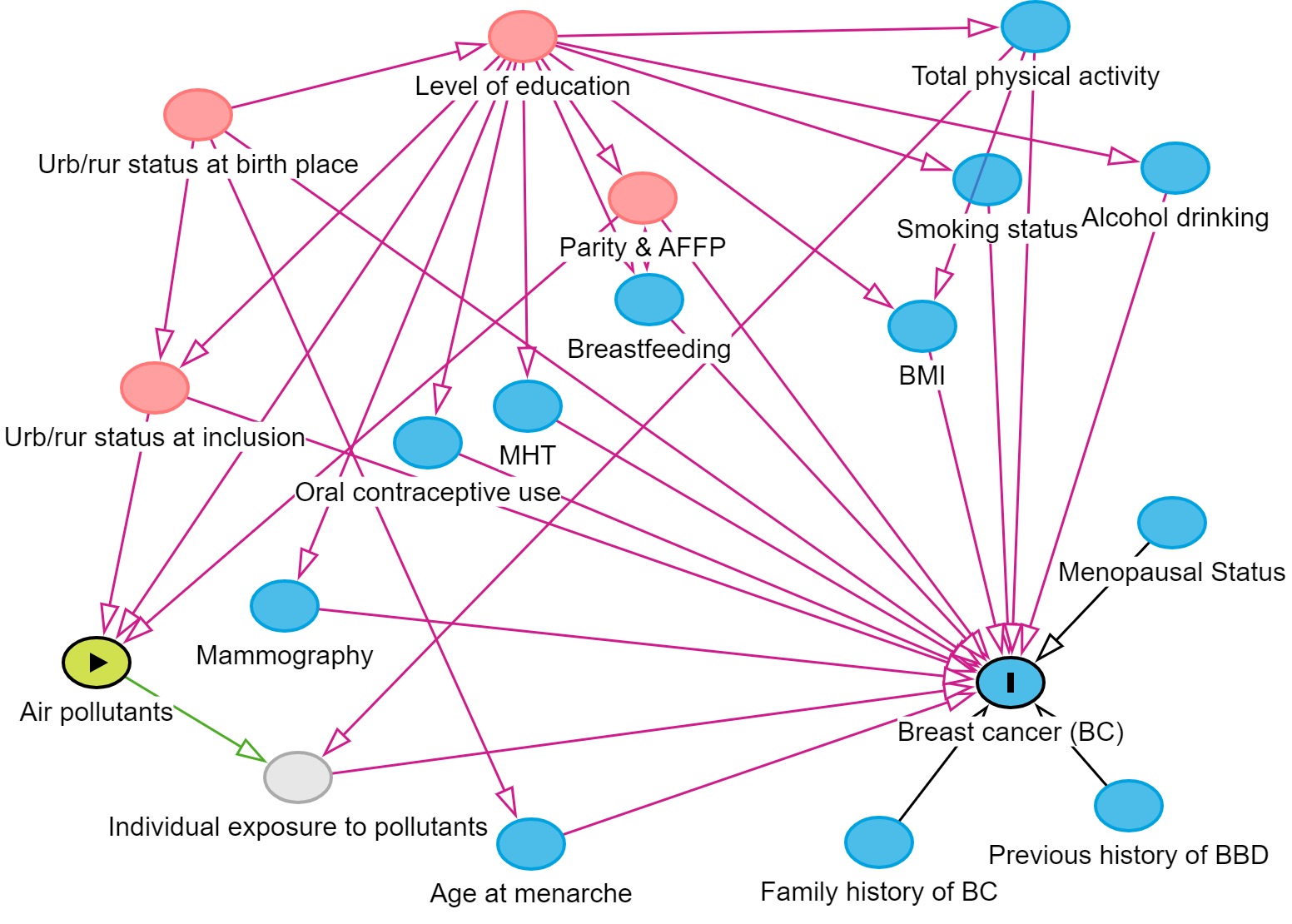
**

**Supplementary Figure 2: Directed Acyclic Graph showing the relationship between potential confounders in the association between air pollutants and breast cancer risk.**

BBD: benign breast disease, MHT: menopause hormone treatment, BMI: body mass index, AFFP: age at first full-term pregnancy

**Supplementary Table 1:** Demographic and lifestyle characteristics of breast cancer cases and matched controls in a nested case-control study within the French E3N-Generations cohort,1990-2011

| **Characteristics** | **Cases (523)** | **Controls (523)** | **Total** |
| --- | --- | --- | --- |
| Age at inclusion (years) |  |  |  |
| Mean (SD) | 49.91 (6.3) | 49.9 (6.3) | 49.9 (6.3) |
| Median | 49.1 | 49.0 | 49.0 |
| Q1-Q3 | 44.3 - 54.7 | 44.5 - 54.7 | 44.4 - 54.7 |
| Min-Max | 40.1 - 64.7 | 40.2 - 64.4 | 40.1 - 64.7 |
| Body Mass Index (kg/m²), n (%) |  |  |  |
| < 25 | 459 (87.8%) | 436 (83.4%) | 895 (85.6%) |
| [25-30[ | 50 (9.6%) | 71 (13.6%) | 121 (11.6%) |
| ≥ 30 | 14 (2.7%) | 16 (3.1%) | 30 (2.9%) |
| Physical activity (METs-h/week), n (%) |  |  |  |
| <25.3 | 121 (23.1%) | 128 (24.5%) | 249 (23.8%) |
| 25.3-35.6 | 156 (29.8%) | 151 (28.9%) | 307 (29.3%) |
| 35.6-51.8 | 130 (24.9%) | 125 (23.9%) | 255 (24.4%) |
| ≥51.8 | 116 (22.2%) | 119 (22.8%) | 235 (22.5%) |
| Alcohol consumption (g/day), n (%) |  |  |  |
| 0 | 58 (11.1%) | 50 (9.6%) | 108 (10.3%) |
| 0-6.7 | 193 (36.9%) | 179 (34.2%) | 372 (35.6%) |
| >6.7 | 272 (52.0%) | 294 (56.2%) | 566 (54.1%) |
| Smoking status, n (%) |  |  |  |
| Non-smoker | 280 (53.5%) | 274 (52.4%) | 554 (53.0%) |
| Smoker | 71 (13.6%) | 73 (14.0%) | 144 (13.8%) |
| Ex-smoker | 172 (32.9%) | 176 (33.7%) | 348 (33.3%) |
| Level of education, n (%) |  |  |  |
| Secondary | 72 (13.8%) | 67 (12.8%) | 139 (13.3%) |
| 1- to 2-year university degree | 231 (44.2%) | 231 (44.2%) | 462 (44.2%) |
| ≥ 3-year university degree | 220 (42.1%) | 225 (43.0%) | 445 (42.5%) |
| Age at first menstruation (years), n (%) |  |  |  |
| <12 | 103 (19.7%) | 102 (19.5%) | 205 (19.6%) |
| 12-14 | 263 (50.3%) | 287 (54.9%) | 550 (52.6%) |
| ≥14 | 157 (30.0%) | 134 (25.6%) | 291 (27.8%) |
| Number of children and age at 1st pregnancy, n (%) |  |  |  |
| No children | 62 (11.9%) | 77 (14.7%) | 139 (13.3%) |
| Between 0 and 2 and age < 30 y.o | 238 (45.5%) | 234 (44.7%) | 472 (45.1%) |
| Between 0 and 2 and age ≥ 30 y.o | 52 (9.9%) | 53 (10.1%) | 105 (10.0%) |
| More than 3 y.o | 171 (32.7%) | 159 (30.4%) | 330 (31.5%) |
| Breastfeeding, n (%) |  |  |  |
| No | 196 (37.5%) | 214 (40.9%) | 410 (39.2%) |
| Yes | 327 (62.5%) | 309 (59.1%) | 636 (60.8%) |
| Use of oral contraceptives until the index date, n (%) |  |  |  |
| No | 207 (39.6%) | 217 (41.5%) | 424 (40.5%) |
| Yes | 316 (60.4%) | 306 (58.5%) | 622 (59.5%) |
| Personal history of benign breast disease at inclusion, n (%) |  |  |  |
| No | 394 (75.3%) | 384 (73.4%) | 778 (74.4%) |
| Yes | 129 (24.7%) | 139 (26.6%) | 268 (25.6%) |
| Family history at inclusion, n (%) |  |  |  |
| No | 454 (86.8%) | 419 (80.1%) | 873 (83.5%) |
| Yes | 69 (13.2%) | 104 (19.9%) | 173 (16.5%) |
| Mammography performed before inclusion, n (%) |  |  |  |
| No | 111 (21.2%) | 101 (19.3%) | 212 (20.3%) |
| Yes | 412 (78.8%) | 422 (80.7%) | 834 (79.7%) |
| Menopausal status at inclusion, n (%) |  |  |  |
| Premenopausal | 306 (58.5%) | 293 (56.0%) | 599 (57.3%) |
| Postmenopausal | 217 (41.5%) | 230 (44.0%) | 447 (42.7%) |
| Menopausal status at index date, n (%) |  |  |  |
| Premenopausal | 38 (7.3%) | 48 (9.2%) | 86 (8.2%) |
| Postmenopausal | 485 (92.7%) | 475 (90.8%) | 960 (91.8%) |
| Menopause hormone treatment (MHT), n (%) |  |  |  |
| No | 146 (27.9%) | 135 (25.8%) | 281 (26.9%) |
| Yes | 363 (69.4%) | 381 (72.8%) | 744 (71.1%) |
| Missing | 14 (2.7%) | 7 (1.3%) | 21 (2.0%) |
| Urban-rural status at birth, n (%) |  |  |  |
| Rural | 123 (23.5%) | 117 (22.4%) | 240 (22.9%) |
| Urban | 343 (65.6%) | 355 (67.9%) | 698 (66.7%) |
| Missing | 57 (10.9%) | 51 (9.8%) | 108 (10.3%) |
| Urban-rural status at inclusion, n (%) |  |  |  |
| Rural | 103 (19.7%) | 98 (18.7%) | 201 (19.2%) |
| Urban | 420 (80.3%) | 425 (81.3%) | 845 (80.8%) |

SD: standard deviation, Q1: 1st quartile, Q3: 3rd quartile, y.o: years old

**Supplementary Table 2:** Biomarker levels of breast cancer cases and matched controls in a nested case-control study within the French E3N-Generations cohort, 1990-2011

| **Biomarkers** | **Cases (523)** | **Controls (523)** | **Total (1046)** |
| --- | --- | --- | --- |
| Albumin |  |  |  |
| Mean (SD) | 41.57 (3.48) | 41.44 (3.53) | 41.50 (3.50) |
| Median | 42.00 | 42.00 | 42.00 |
| Q1-Q3 | 40.00 - 44.00 | 39.00 - 44.00 | 39.00 - 44.00 |
| Min-Max | 27.00 - 58.00 | 28.00 - 51.00 | 27.00 - 58.00 |
| Missing | 18 | 27 | 45 |
| Protein C-reactive |  |  |  |
| Mean (SD) | 0.86 (0.53) | 0.85 (0.59) | 0.86 (0.56) |
| Median | 0.69 | 0.69 | 0.69 |
| Q1-Q3 | 0.69 - 1.10 | 0.69 - 1.10 | 0.69 - 1.10 |
| Min-Max | 0.00 - 3.37 | 0.00 - 3.83 | 0.00 - 3.83 |
| Missing | 19 | 24 | 43 |
| Triglycerides |  |  |  |
| Mean (SD) | 0.68 (0.21) | 0.68 (0.21) | 0.68 (0.21) |
| Median | 0.64 | 0.65 | 0.65 |
| Q1-Q3 | 0.54 - 0.78 | 0.54 - 0.78 | 0.54 - 0.78 |
| Min-Max | 0.28 - 1.88 | 0.10 - 1.65 | 0.10 - 1.88 |
| Missing | 44 | 57 | 101 |
| Cholesterol |  |  |  |
| Mean (SD) | 5.22 (0.85) | 5.25 (0.83) | 5.24 (0.84) |
| Median | 5.22 | 5.20 | 5.21 |
| Q1-Q3 | 4.66 - 5.76 | 4.67 - 5.72 | 4.66 - 5.74 |
| Min-Max | 2.35 - 8.25 | 2.86 - 8.08 | 2.35 - 8.25 |
| Missing | 34 | 40 | 74 |
| HDL cholesterol |  |  |  |
| Mean (SD) | 1.15 (0.26) | 1.15 (0.27) | 1.15 (0.27) |
| Median | 1.11 | 1.11 | 1.11 |
| Q1-Q3 | 0.95 - 1.36 | 0.95 - 1.36 | 0.95 - 1.36 |
| Min-Max | 0.43 - 1.81 | 0.55 - 1.75 | 0.43 - 1.81 |
| Missing | 53 | 57 | 110 |
| LDL cholesterol |  |  |  |
| Mean (SD) | 1.19 (0.27) | 1.20 (0.26) | 1.20 (0.27) |
| Median | 1.18 | 1.21 | 1.20 |
| Q1-Q3 | 0.97 - 1.42 | 1.00 - 1.40 | 0.98 - 1.41 |
| Min-Max | 0.55 - 1.82 | 0.31 - 1.87 | 0.31 - 1.87 |
| Missing | 47 | 62 | 109 |
| Parathormone |  |  |  |
| Mean (SD) | 1.26 (0.28) | 1.27 (0.26) | 1.27 (0.27) |
| Median | 1.25 | 1.27 | 1.26 |
| Q1-Q3 | 1.08 - 1.41 | 1.10 - 1.43 | 1.09 - 1.43 |
| Min-Max | 0.44 - 2.42 | 0.48 - 2.16 | 0.44 - 2.42 |
| Missing | 23 | 31 | 54 |
| TSH |  |  |  |
| Mean (SD) | 0.93 (0.39) | 0.98 (0.41) | 0.95 (0.40) |
| Median | 0.89 | 0.93 | 0.91 |
| Q1-Q3 | 0.68 - 1.13 | 0.74 - 1.17 | 0.71 - 1.15 |
| Min-Max | 0.01 - 4.06 | 0.01 - 4.13 | 0.01 - 4.13 |
| Missing | 22 | 21 | 43 |
| Prolactin |  |  |  |
| Mean (SD) | 5.31 (0.50) | 5.30 (0.51) | 5.31 (0.51) |
| Median | 5.28 | 5.28 | 5.28 |
| Q1-Q3 | 5.01 - 5.60 | 4.97 - 5.61 | 4.99 - 5.60 |
| Min-Max | 2.87 - 8.33 | 4.17 - 8.17 | 2.87 - 8.33 |
| Missing | 16 | 23 | 39 |
| Estradiol |  |  |  |
| Mean (SD) | 4.98 (1.20) | 4.82 (1.20) | 4.90 (1.20) |
| Median | 5.06 | 4.83 | 4.98 |
| Q1-Q3 | 4.20 - 5.80 | 3.88 - 5.70 | 4.04 - 5.76 |
| Min-Max | 2.96 - 8.27 | 2.96 - 9.25 | 2.96 - 9.25 |
| Missing | 18 | 26 | 44 |
| Testosterone |  |  |  |
| Mean (SD) | 0.68 (0.40) | 0.69 (0.40) | 0.69 (0.40) |
| Median | 0.64 | 0.65 | 0.65 |
| Q1-Q3 | 0.42 - 0.90 | 0.44 - 0.89 | 0.43 - 0.90 |
| Min-Max | 0.07 - 3.51 | 0.07 - 3.72 | 0.07 - 3.72 |
| Missing | 23 | 30 | 53 |
| SHBG |  |  |  |
| Mean (SD) | 4.07 (0.48) | 4.11 (0.50) | 4.09 (0.49) |
| Median | 4.10 | 4.09 | 4.09 |
| Q1-Q3 | 3.74 - 4.38 | 3.80 - 4.44 | 3.76 - 4.42 |
| Min-Max | 2.59 - 5.30 | 1.91 - 5.45 | 1.91 - 5.45 |
| Missing | 36 | 50 | 86 |
| Progesterone |  |  |  |
| Mean (SD) | 1.05 (1.02) | 1.00 (0.94) | 1.03 (0.98) |
| Median | 0.67 | 0.66 | 0.67 |
| Q1-Q3 | 0.44 - 1.04 | 0.44 - 1.04 | 0.44 - 1.04 |
| Min-Max | 0.00 - 5.24 | 0.00 - 4.67 | 0.00 - 5.24 |
| Missing | 17 | 25 | 42 |

SD: standard deviation, Q1: 1st quartile, Q3: 3rd quartile, HDL cholesterol: High-density lipoprotein cholesterol, LDL cholesterol: Light-density lipoprotein cholesterol, TSH: Thyroid-stimulating hormone, SHGB: Sex Hormone-Binding Globulin.

**Supplementary Table 3:** Pollutant levels exposition of breast cancer cases and matched controls in a nested case-control study within the French E3N-Generations cohort, 1990-2011

| **Pollutants** | **Cases (523)** | **Controls (523)** | **Total (1046)** |
| --- | --- | --- | --- |
| NO_2_ |  |  |  |
| Mean (SD) | 37.31 (16.91) | 36.85 (16.99) | 37.08 (16.94) |
| Median | 32.93 | 32.44 | 32.78 |
| Q1-Q3 | 25.09 - 46.87 | 24.21 - 46.47 | 24.58 - 46.64 |
| Min-Max | 9.93 - 91.41 | 10.89 - 94.14 | 9.93 - 94.14 |
| BaP |  |  |  |
| Mean (SD) | 0.21 (0.12) | 0.21 (0.13) | 0.21 (0.12) |
| Median | 0.18 | 0.18 | 0.18 |
| Q1-Q3 | 0.14 - 0.24 | 0.13 - 0.24 | 0.14 - 0.24 |
| Min-Max | 0.07 - 1.12 | 0.06 - 1.12 | 0.06 - 1.12 |
| PCB153 |  |  |  |
| Mean (SD) | 11.17 (4.15) | 10.95 (3.92) | 11.06 (4.04) |
| Median | 10.56 | 10.12 | 10.39 |
| Q1-Q3 | 8.19 - 12.94 | 8.13 - 12.91 | 8.16 - 12.93 |
| Min-Max | 3.17 - 24.96 | 3.83 - 24.96 | 3.17 - 24.96 |

SD: standard deviation, Q1: 1st quartile, Q3: 3rd quartile, NO_2_: nitrogen dioxide, BaP: benzo[a]pyrene, PCB153: polychlorinated biphenyls

**Supplementary Table 4**: Beta coefficient (CI95%) and proportion attributable for the four-way decomposition of each mediator of the associations between NO_2_ exposure from inclusion to biomarker collection date and breast cancer risk, a nested case-control study within the E3N-Generations cohort, 1990-2011

| **Mediation** | **Effect** | **Estimate (CI 95%)** | **P value** | **Proportion** | **P value** |
| --- | --- | --- | --- | --- | --- |
| Albumin | TE | 0.1015 (-0.2914, 0.4945) | 0.613 |  |  |
|  | CDE | 0.0950 (-0.2990, 0.4890) | 0.636 | 93.6% | <0.001 |
|  | INTref | 0.0046 (-0.0257, 0.0349) | 0.766 | 4.5% | 0.799 |
|  | INTmed | -0.0021 (-0.0159, 0.0117) | 0.766 | -2.1% | 0.798 |
|  | PIE | 0.0040 (-0.0103, 0.0184) | 0.583 | 4.0% | 0.708 |
|  | O_M |  |  | 1.9% | 0.795 |
| CRP | TE | 0.1032 (-0.2878, 0.4943) | 0.605 |  |  |
|  | CDE | 0.1171 (-0.2719, 0.5061) | 0.555 | 113.4% | 0.006 |
|  | INTref | -0.0136 (-0.0728, 0.0456) | 0.652 | -13.2% | 0.747 |
|  | INTmed | 0.0004 (-0.0056, 0.0065) | 0.893 | 0.4% | 0.897 |
|  | PIE | -0.0007 (-0.0099, 0.0086) | 0.889 | -0.6% | 0.894 |
|  | O_M |  |  | -0.2% | 0.905 |
| Triglycerides | TE | 0.1158 (-0.2967, 0.5284) | 0.582 |  |  |
|  | CDE | 0.1127 (-0.3024, 0.5277) | 0.595 | 97.3% | <0.001 |
|  | INTref | 0.0003 (-0.0309, 0.0315) | 0.984 | 0.3% | 0.984 |
|  | INTmed | -0.0002 (-0.0118, 0.0115) | 0.979 | -0.1% | 0.979 |
|  | PIE | 0.0030 (-0.0102, 0.0161) | 0.657 | 2.6% | 0.725 |
|  | O_M |  |  | 2.4% | 0.715 |
| Cholesterol | TE | 0.0742 (-0.3160, 0.4643) | 0.710 |  |  |
|  | CDE | 0.0733 (-0.3173, 0.4639) | 0.713 | 98.8% | <0.001 |
|  | INTref | -0.0010 (-0.0113, 0.0093) | 0.843 | -1.4% | 0.855 |
|  | INTmed | 0.0012 (-0.0074, 0.0099) | 0.777 | 1.7% | 0.812 |
|  | PIE | 0.0006 (-0.0048, 0.0061) | 0.817 | 0.9% | 0.844 |
|  | O_M |  |  | 2.6% | 0.807 |
| HDL cholesterol | TE | 0.1465 (-0.2836, 0.5766) | 0.504 |  |  |
|  | CDE | 0.1482 (-0.2854, 0.5818) | 0.503 | 101.2% | <0.001 |
|  | INTref | -0.0064 (-0.0423, 0.0296) | 0.728 | -4.4% | 0.747 |
|  | INTmed | 0.0027 (-0.0137, 0.0191) | 0.749 | 1.8% | 0.764 |
|  | PIE | 0.0020 (-0.0142, 0.0182) | 0.808 | 1.4% | 0.819 |
|  | O_M |  |  | 3.2% | 0.693 |
| LDL cholesterol | TE | 0.2345 (-0.2479, 0.7169) | 0.341 |  |  |
|  | CDE | 0.2292 (-0.2396, 0.6980) | 0.338 | 97.7% | <0.001 |
|  | INTref | 0.0199 (-0.0383, 0.0782) | 0.502 | 8.5% | 0.479 |
|  | INTmed | -0.0094 (-0.0377, 0.0189) | 0.515 | -4.0% | 0.510 |
|  | PIE | -0.0053 (-0.0254, 0.0148) | 0.608 | -2.2% | 0.657 |
|  | O_M |  |  | -6.2% | 0.487 |
| Parathormone | TE | 0.1273 (-0.2893, 0.5439) | 0.549 |  |  |
|  | CDE | 0.1131 (-0.2981, 0.5242) | 0.590 | 88.8% | <0.001 |
|  | INTref | -0.0060 (-0.0248, 0.0128) | 0.533 | -4.7% | 0.634 |
|  | INTmed | 0.0118 (-0.0206, 0.0442) | 0.475 | 9.3% | 0.607 |
|  | PIE | 0.0084 (-0.0187, 0.0356) | 0.544 | 6.6% | 0.648 |
|  | O_M |  |  | 15.9% | 0.542 |
| TSH | TE | 0.0447 (-0.3367, 0.4261) | 0.818 |  |  |
|  | CDE | 0.0447 (-0.3397, 0.4291) | 0.820 | 99.9% | 0.024 |
|  | INTref | 0.0016 (-0.0296, 0.0329) | 0.919 | 3.6% | 0.927 |
|  | INTmed | 0.0044 (-0.0214, 0.0302) | 0.737 | 9.9% | 0.856 |
|  | PIE | -0.0060 (-0.0406, 0.0286) | 0.734 | -13.4% | 0.853 |
|  | O_M |  |  | -3.6% | 0.853 |
| Prolactin | TE | 0.1410 (-0.2698, 0.5518) | 0.501 |  |  |
|  | CDE | 0.1436 (-0.2692, 0.5563) | 0.495 | 101.8% | <0.001 |
|  | INTref | 0.0028 (-0.0148, 0.0204) | 0.752 | 2.0% | 0.768 |
|  | INTmed | -0.0122 (-0.0404, 0.0160) | 0.396 | -8.6% | 0.560 |
|  | PIE | 0.0068 (-0.0161, 0.0296) | 0.560 | 4.8% | 0.645 |
|  | O_M |  |  | -3.8% | 0.704 |
| Estradiol | TE | 0.0744 (-0.3118, 0.4606) | 0.706 |  |  |
|  | CDE | 0.0580 (-0.3271, 0.4431) | 0.768 | 78.0% | 0.196 |
|  | INTref | -0.0013 (-0.0134, 0.0107) | 0.827 | -1.8% | 0.800 |
|  | INTmed | -0.0049 (-0.0259, 0.0162) | 0.649 | -6.6% | 0.798 |
|  | PIE | 0.0226 (-0.0130, 0.0581) | 0.214 | 30.3% | 0.713 |
|  | O_M |  |  | 23.8% | 0.695 |
| Testosterone | TE | 0.1023 (-0.3084, 0.5129) | 0.626 |  |  |
|  | CDE | 0.1266 (-0.2845, 0.5378) | 0.546 | 123.8% | 0.022 |
|  | INTref | -0.0179 (-0.0697, 0.0339) | 0.499 | -17.5% | 0.708 |
|  | INTmed | -0.0147 (-0.0542, 0.0249) | 0.468 | -14.3% | 0.669 |
|  | PIE | 0.0081 (-0.0148, 0.0311) | 0.487 | 8.0% | 0.692 |
|  | O_M |  |  | -6.4% | 0.664 |
| SHBG | TE | 0.1257 (-0.2945, 0.5460) | 0.558 |  |  |
|  | CDE | 0.1251 (-0.2951, 0.5454) | 0.560 | 99.5% | <0.001 |
|  | INTref | 0.0004 (-0.0061, 0.0068) | 0.910 | 0.3% | 0.912 |
|  | INTmed | -0.0004 (-0.0054, 0.0045) | 0.860 | -0.4% | 0.866 |
|  | PIE | 0.0007 (-0.0061, 0.0075) | 0.841 | 0.6% | 0.850 |
|  | O_M |  |  | 0.2% | 0.894 |
| Progesterone | TE | 0.0890 (-0.3027, 0.4808) | 0.656 |  |  |
|  | CDE | 0.0697 (-0.3233, 0.4627) | 0.728 | 78.3% | 0.221 |
|  | INTref | 0.0211 (-0.0606, 0.1028) | 0.613 | 23.7% | 0.727 |
|  | INTmed | -0.0010 (-0.0088, 0.0069) | 0.807 | -1.1% | 0.829 |
|  | PIE | -0.0008 (-0.0073, 0.0057) | 0.810 | -0.9% | 0.832 |
|  | O_M |  |  | -2.0% | 0.817 |

TE: total effect (total excess relative risk), CDE: excess relative risk due to controlled direct effect, INTref: excess relative risk due to reference interaction, INTmed: excess relative risk due to mediated interaction, PIE: excess relative risk due to pure indirect effect, O_M: overall mediated.

CRP: C-reactive protein, HDL: High-density lipoprotein cholesterol, LDL: Light-density lipoprotein cholesterol, TSH: Thyroid-stimulating hormone, SHGB: Sex Hormone-Binding Globulin.

Output of mediation analysis with causal effects estimated for a change in pollutant levels from the 25th to the 75th percentile.

Controlled direct effects are computed fixing the mediators at their median levels.

Adjusted for body mass index, menopausal hormone replacement therapy uses, urban/rural status at birth, urban/rural status at inclusion, alcohol drinking, breastfeeding, and mammography before inclusion, oral contraceptive use, age at full-term pregnancy and parity, smoking status, total physical activity.

**Supplementary Table 5**: Beta coefficient (CI 95%) and proportion attributable for the four-way decomposition of each mediator of the associations between PCB153 exposure from inclusion to biomarker collection date and breast cancer risk, a nested case-control study within the E3N-Generations cohort, 1990-2011

| **Mediation** | **Effect** | **Estimate (CI 95%)** | **P value** | **Proportion** | **P value** |
| --- | --- | --- | --- | --- | --- |
| Albumin | TE | 0.4596 (-0.1065, 1.0257) | 0.112 |  |  |
|  | CDE | 0.4394 (-0.1250, 1.0038) | 0.127 | 95.6% | <0.001 |
|  | INTref | 0.0210 (-0.0358, 0.0777) | 0.469 | 4.6% | 0.500 |
|  | INTmed | -0.0160 (-0.0530, 0.0210) | 0.397 | -3.5% | 0.440 |
|  | PIE | 0.0152 (-0.0137, 0.0442) | 0.303 | 3.3% | 0.374 |
|  | O_M |  |  | -0.2% | 0.968 |
| CRP | TE | 0.4050 (-0.1396, 0.9496) | 0.145 |  |  |
|  | CDE | 0.4408 (-0.1139, 0.9955) | 0.119 | 108.8% | <0.001 |
|  | INTref | -0.0350 (-0.0974, 0.0274) | 0.271 | -8.7% | 0.363 |
|  | INTmed | -0.0047 (-0.0219, 0.0125) | 0.594 | -1.2% | 0.608 |
|  | PIE | 0.0039 (-0.0104, 0.0182) | 0.593 | 1.0% | 0.618 |
|  | O_M |  |  | -0.2% | 0.870 |
| Triglycerides | TE | 0.4762 (-0.1145, 1.0670) | 0.114 |  |  |
|  | CDE | 0.4916 (-0.1061, 1.0892) | 0.107 | 103.2% | <0.001 |
|  | INTref | -0.0148 (-0.0459, 0.0162) | 0.348 | -3.1% | 0.376 |
|  | INTmed | -0.0005 (-0.0137, 0.0127) | 0.938 | -0.1% | 0.938 |
|  | PIE | 0.0001 (-0.0009, 0.0010) | 0.978 | 0.0% | 0.978 |
|  | O_M |  |  | -0.1% | 0.938 |
| Cholesterol | TE | 0.5158 (-0.0893, 1.1209) | 0.095 |  |  |
|  | CDE | 0.5197 (-0.0873, 1.1267) | 0.093 | 100.8% | <0.001 |
|  | INTref | -0.0008 (-0.0217, 0.0201) | 0.943 | -0.1% | 0.943 |
|  | INTmed | -0.0033 (-0.0198, 0.0131) | 0.691 | -0.6% | 0.693 |
|  | PIE | 0.0002 (-0.0047, 0.0051) | 0.926 | 0.0% | 0.926 |
|  | O_M |  |  | -0.6% | 0.700 |
| HDL cholesterol | TE | 0.5116 (-0.0974, 1.1205) | 0.100 |  |  |
|  | CDE | 0.5268 (-0.0918, 1.1454) | 0.095 | 103.0% | <0.001 |
|  | INTref | -0.0087 (-0.0361, 0.0186) | 0.532 | -1.7% | 0.525 |
|  | INTmed | -0.0066 (-0.0288, 0.0156) | 0.559 | -1.3% | 0.558 |
|  | PIE | 0.0001 (-0.0164, 0.0167) | 0.988 | 0.0% | 0.988 |
|  | O_M |  |  | -1.3% | 0.626 |
| LDL cholesterol | TE | 0.6204 (-0.0535, 1.2943) | 0.071 |  |  |
|  | CDE | 0.5838 (-0.0607, 1.2282) | 0.076 | 94.1% | <0.001 |
|  | INTref | 0.0196 (-0.0470, 0.0862) | 0.564 | 3.2% | 0.543 |
|  | INTmed | 0.0141 (-0.0242, 0.0523) | 0.471 | 2.3% | 0.451 |
|  | PIE | 0.0030 (-0.0124, 0.0184) | 0.705 | 0.5% | 0.705 |
|  | O_M |  |  | 2.7% | 0.446 |
| Parathormone | TE | 0.3755 (-0.1758, 0.9268) | 0.182 |  |  |
|  | CDE | 0.3651 (-0.1829, 0.9131) | 0.192 | 97.2% | <0.001 |
|  | INTref | -0.0078 (-0.0268, 0.0112) | 0.422 | -2.1% | 0.446 |
|  | INTmed | 0.0120 (-0.0158, 0.0397) | 0.397 | 3.2% | 0.428 |
|  | PIE | 0.0062 (-0.0165, 0.0289) | 0.590 | 1.7% | 0.603 |
|  | O_M |  |  | 4.9% | 0.330 |
| TSH | TE | 0.3273 (-0.1801, 0.8348) | 0.206 |  |  |
|  | CDE | 0.3391 (-0.1744, 0.8526) | 0.196 | 103.6% | <0.001 |
|  | INTref | -0.0070 (-0.0322, 0.0183) | 0.590 | -2.1% | 0.601 |
|  | INTmed | 0.0033 (-0.0105, 0.0171) | 0.641 | 1.0% | 0.682 |
|  | PIE | -0.0081 (-0.0377, 0.0214) | 0.590 | -2.5% | 0.629 |
|  | O_M |  |  | -1.5% | 0.629 |
| Prolactin | TE | 0.4180 (-0.1263, 0.9622) | 0.132 |  |  |
|  | CDE | 0.4185 (-0.1264, 0.9633) | 0.132 | 100.1% | <0.001 |
|  | INTref | 0.0006 (-0.0066, 0.0078) | 0.872 | 0.1% | 0.872 |
|  | INTmed | -0.0031 (-0.0220, 0.0157) | 0.744 | -0.8% | 0.747 |
|  | PIE | 0.0020 (-0.0147, 0.0188) | 0.811 | 0.5% | 0.813 |
|  | O_M |  |  | -0.3% | 0.919 |
| Estradiol | TE | 0.3641 (-0.1632, 0.8915) | 0.176 |  |  |
|  | CDE | 0.3461 (-0.1809, 0.8731) | 0.198 | 95.0% | <0.001 |
|  | INTref | -0.0059 (-0.0179, 0.0060) | 0.330 | -1.6% | 0.123 |
|  | INTmed | 0.0064 (-0.0176, 0.0303) | 0.602 | 1.7% | 0.571 |
|  | PIE | 0.0176 (-0.0109, 0.0462) | 0.226 | 4.8% | 0.355 |
|  | O_M |  |  | 6.6% | 0.288 |
| Testosterone | TE | 0.3384 (-0.2006, 0.8774) | 0.218 |  |  |
|  | CDE | 0.3609 (-0.1789, 0.9007) | 0.190 | 106.6% | <0.001 |
|  | INTref | -0.0168 (-0.0627, 0.0292) | 0.474 | -5.0% | 0.555 |
|  | INTmed | -0.0145 (-0.0474, 0.0183) | 0.386 | -4.3% | 0.453 |
|  | PIE | 0.0088 (-0.0124, 0.0301) | 0.414 | 2.6% | 0.503 |
|  | O_M |  |  | -1.7% | 0.554 |
| SHBG | TE | 0.4800 (-0.1327, 1.0927) | 0.125 |  |  |
|  | CDE | 0.4780 (-0.1347, 1.0907) | 0.126 | 99.6% | <0.001 |
|  | INTref | 0.0016 (-0.0151, 0.0183) | 0.850 | 0.3% | 0.851 |
|  | INTmed | -0.0034 (-0.0169, 0.0100) | 0.619 | -0.7% | 0.633 |
|  | PIE | 0.0038 (-0.0088, 0.0164) | 0.557 | 0.8% | 0.578 |
|  | O_M |  |  | 0.1% | 0.949 |
| Progesterone | TE | 0.4074 (-0.1327, 0.9475) | 0.139 |  |  |
|  | CDE | 0.4122 (-0.1267, 0.9511) | 0.134 | 101.2% | <0.001 |
|  | INTref | -0.0080 (-0.0920, 0.0760) | 0.851 | -2.0% | 0.854 |
|  | INTmed | -0.0009 (-0.0113, 0.0096) | 0.870 | -0.2% | 0.872 |
|  | PIE | 0.0041 (-0.0095, 0.0178) | 0.555 | 1.0% | 0.579 |
|  | O_M |  |  | 0.8% | 0.636 |

TE: total effect (total excess relative risk), CDE: excess relative risk due to controlled direct effect, INTref: excess relative risk due to reference interaction, INTmed: excess relative risk due to mediated interaction, PIE: excess relative risk due to pure indirect effect, O_M: overall mediated.

CRP: C-reactive protein, HDL: High-density lipoprotein cholesterol, LDL: Light-density lipoprotein cholesterol, TSH: Thyroid-stimulating hormone, SHGB: Sex Hormone-Binding Globulin.

Output of mediation analysis with causal effects estimated for a change in pollutant levels from the 25th to the 75th percentile.

Controlled direct effects are computed fixing the mediators at their median levels.

Adjusted for body mass index, menopausal hormone replacement therapy uses, urban/rural status at birth, urban/rural status at inclusion, alcohol drinking, breastfeeding, and mammography before inclusion, oral contraceptive use, age at full-term pregnancy and parity, smoking status, total physical activity.

**Supplementary Table 6**: Beta coefficient (CI95%) and proportion attributable for the four-way decomposition of each mediator of the associations between BaP exposure from inclusion to biomarker collection date and breast cancer risk, a nested case-control study within the E3N-Generations cohort, 1990-2011

| **Mediation** | **Effect** | **Estimate (CI 95%)** | **P value** | **Proportion** | **P value** |
| --- | --- | --- | --- | --- | --- |
| Albumin | TE | 0.0153 (-0.2241, 0.2547) | 0.900 |  |  |
|  | CDE | 0.0109 (-0.2284, 0.2503) | 0.929 | 71.5% | 0.760 |
|  | INTref | 0.0005 (-0.0202, 0.0212) | 0.962 | 3.3% | 0.964 |
|  | INTmed | -0.0003 (-0.0111, 0.0105) | 0.961 | -1.7% | 0.963 |
|  | PIE | 0.0041 (-0.0105, 0.0187) | 0.580 | 26.9% | 0.902 |
|  | O_M |  |  | 25.2% | 0.902 |
| CRP | TE | 0.0427 (-0.2201, 0.3055) | 0.750 |  |  |
|  | CDE | 0.0653 (-0.1908, 0.3214) | 0.617 | 152.8% | 0.430 |
|  | INTref | -0.0215 (-0.0814, 0.0385) | 0.483 | -50.3% | 0.789 |
|  | INTmed | 0.0023 (-0.0058, 0.0104) | 0.571 | 5.5% | 0.798 |
|  | PIE | -0.0034 (-0.0134, 0.0065) | 0.499 | -8.1% | 0.779 |
|  | O_M |  |  | -2.6% | 0.811 |
| Triglycerides | TE | 0.0348 (-0.2261, 0.2956) | 0.794 |  |  |
|  | CDE | 0.0286 (-0.2311, 0.2882) | 0.829 | 82.1% | 0.251 |
|  | INTref | 0.0058 (-0.0139, 0.0255) | 0.563 | 16.7% | 0.804 |
|  | INTmed | -0.0006 (-0.0049, 0.0037) | 0.785 | -1.7% | 0.845 |
|  | PIE | 0.0010 (-0.0059, 0.0080) | 0.773 | 2.9% | 0.844 |
|  | O_M |  |  | 1.2% | 0.859 |
| Cholesterol | TE | 0.0737 (-0.2066, 0.3541) | 0.606 |  |  |
|  | CDE | 0.0769 (-0.2044, 0.3582) | 0.592 | 104.4% | <0.001 |
|  | INTref | 0.0008 (-0.0170, 0.0186) | 0.927 | 1.1% | 0.927 |
|  | INTmed | -0.0047 (-0.0153, 0.0059) | 0.386 | -6.4% | 0.631 |
|  | PIE | 0.0006 (-0.0071, 0.0084) | 0.873 | 0.9% | 0.877 |
|  | O_M |  |  | -5.5% | 0.647 |
| HDL cholesterol | TE | 0.0631 (-0.2286, 0.3547) | 0.672 |  |  |
|  | CDE | 0.0839 (-0.2172, 0.3850) | 0.585 | 133.0% | 0.070 |
|  | INTref | -0.0030 (-0.0381, 0.0321) | 0.869 | -4.7% | 0.875 |
|  | INTmed | -0.0229 (-0.0561, 0.0104) | 0.178 | -36.3% | 0.651 |
|  | PIE | 0.0050 (-0.0311, 0.0411) | 0.786 | 7.9% | 0.807 |
|  | O_M |  |  | -28.3% | 0.680 |
| LDL cholesterol | TE | 0.0528 (-0.2144, 0.3201) | 0.698 |  |  |
|  | CDE | 0.0373 (-0.2242, 0.2989) | 0.780 | 70.7% | 0.350 |
|  | INTref | 0.0008 (-0.0080, 0.0096) | 0.858 | 1.5% | 0.870 |
|  | INTmed | 0.0035 (-0.0184, 0.0253) | 0.756 | 6.6% | 0.791 |
|  | PIE | 0.0112 (-0.0173, 0.0397) | 0.440 | 21.2% | 0.712 |
|  | O_M |  |  | 27.8% | 0.690 |
| Parathormone | TE | 0.0390 (-0.2079, 0.2860) | 0.757 |  |  |
|  | CDE | 0.0371 (-0.2089, 0.2831) | 0.768 | 95.0% | 0.001 |
|  | INTref | -0.0018 (-0.0163, 0.0128) | 0.810 | -4.6% | 0.850 |
|  | INTmed | 0.0027 (-0.0052, 0.0105) | 0.510 | 6.8% | 0.766 |
|  | PIE | 0.0011 (-0.0048, 0.0070) | 0.716 | 2.8% | 0.807 |
|  | O_M |  |  | 9.6% | 0.764 |
| TSH | TE | 0.0397 (-0.2082, 0.2876) | 0.754 |  |  |
|  | CDE | 0.0361 (-0.2146, 0.2868) | 0.778 | 91.0% | 0.012 |
|  | INTref | 0.0005 (-0.0071, 0.0081) | 0.897 | 1.3% | 0.918 |
|  | INTmed | -0.0007 (-0.0046, 0.0033) | 0.747 | -1.7% | 0.834 |
|  | PIE | 0.0037 (-0.0101, 0.0176) | 0.595 | 9.4% | 0.782 |
|  | O_M |  |  | 7.8% | 0.776 |
| Prolactin | TE | 0.0284 (-0.2159, 0.2728) | 0.820 |  |  |
|  | CDE | 0.0263 (-0.2171, 0.2696) | 0.832 | 92.4% | 0.025 |
|  | INTref | 0.0011 (-0.0111, 0.0133) | 0.860 | 3.9% | 0.884 |
|  | INTmed | 0.0022 (-0.0047, 0.0090) | 0.535 | 7.6% | 0.830 |
|  | PIE | -0.0011 (-0.0066, 0.0044) | 0.691 | -3.9% | 0.847 |
|  | O_M |  |  | 3.7% | 0.838 |
| Estradiol | TE | 0.0411 (-0.2065, 0.2887) | 0.745 |  |  |
|  | CDE | 0.0294 (-0.2176, 0.2764) | 0.815 | 71.5% | 0.421 |
|  | INTref | -0.0005 (-0.0048, 0.0037) | 0.799 | -1.3% | 0.691 |
|  | INTmed | -0.0001 (-0.0087, 0.0086) | 0.987 | -0.2% | 0.987 |
|  | PIE | 0.0123 (-0.0064, 0.0310) | 0.196 | 30.0% | 0.750 |
|  | O_M |  |  | 29.8% | 0.737 |
| Testosterone | TE | 0.0217 (-0.2249, 0.2683) | 0.863 |  |  |
|  | CDE | 0.0234 (-0.2190, 0.2659) | 0.850 | 108.0% | 0.128 |
|  | INTref | -0.0011 (-0.0202, 0.0180) | 0.910 | -5.1% | 0.933 |
|  | INTmed | 0.0002 (-0.0034, 0.0037) | 0.913 | 0.9% | 0.934 |
|  | PIE | -0.0008 (-0.0056, 0.0040) | 0.735 | -3.8% | 0.883 |
|  | O_M |  |  | -2.9% | 0.877 |
| SHBG | TE | 0.0819 (-0.2007, 0.3645) | 0.570 |  |  |
|  | CDE | 0.0813 (-0.2014, 0.3640) | 0.573 | 99.2% | <0.001 |
|  | INTref | 0.0004 (-0.0095, 0.0103) | 0.944 | 0.4% | 0.944 |
|  | INTmed | -0.0010 (-0.0062, 0.0042) | 0.698 | -1.3% | 0.746 |
|  | PIE | 0.0013 (-0.0051, 0.0076) | 0.692 | 1.6% | 0.742 |
|  | O_M |  |  | 0.3% | 0.872 |
| Progesterone | TE | 0.0321 (-0.2132, 0.2775) | 0.797 |  |  |
|  | CDE | 0.0580 (-0.1924, 0.3085) | 0.650 | 180.7% | 0.582 |
|  | INTref | -0.0281 (-0.0807, 0.0246) | 0.296 | -87.4% | 0.804 |
|  | INTmed | -0.0042 (-0.0139, 0.0054) | 0.387 | -13.2% | 0.805 |
|  | PIE | 0.0064 (-0.0063, 0.0191) | 0.323 | 19.9% | 0.804 |
|  | O_M |  |  | 6.7% | 0.824 |

TE: total effect (total excess relative risk), CDE: excess relative risk due to controlled direct effect, INTref: excess relative risk due to reference interaction, INTmed: excess relative risk due to mediated interaction, PIE: excess relative risk due to pure indirect effect, O_M: overall mediated.

CRP: C-reactive protein, HDL: High-density lipoprotein cholesterol, LDL: Light-density lipoprotein cholesterol, TSH: Thyroid stimulating hormone, SHGB: Sex Hormone-Binding Globulin.

Output of mediation analysis with causal effects estimated for a change in pollutant levels from the 25th to the 75th percentile.

Controlled direct effects are computed fixing the mediators at their median levels.

Adjusted for body mass index, menopausal hormone replacement therapy uses, urban/rural status at birth, urban/rural status at inclusion, alcohol drinking, breastfeeding, and mammography before inclusion, oral contraceptive use, age at full-term pregnancy and parity, smoking status, total physical activity.
